# Insights Into the Role of CSF1R in the Central Nervous System and Neurological Disorders

**DOI:** 10.3389/fnagi.2021.789834

**Published:** 2021-11-15

**Authors:** Banglian Hu, Shengshun Duan, Ziwei Wang, Xin Li, Yuhang Zhou, Xian Zhang, Yun-Wu Zhang, Huaxi Xu, Honghua Zheng

**Affiliations:** ^1^Fujian Provincial Key Laboratory of Neurodegenerative Disease and Aging Research, School of Medicine, Institute of Neuroscience, Xiamen University, Xiamen, China; ^2^Basic Medical Sciences, School of Medicine, Xiamen University, Xiamen, China

**Keywords:** CSF1R, microglia, microglial maintenance, neurogenesis, CSF1R cleavage, CSF1R mutations, ALSP, AD

## Abstract

The colony-stimulating factor 1 receptor (CSF1R) is a key tyrosine kinase transmembrane receptor modulating microglial homeostasis, neurogenesis, and neuronal survival in the central nervous system (CNS). CSF1R, which can be proteolytically cleaved into a soluble ectodomain and an intracellular protein fragment, supports the survival of myeloid cells upon activation by two ligands, colony stimulating factor 1 and interleukin 34. CSF1R loss-of-function mutations are the major cause of adult-onset leukoencephalopathy with axonal spheroids and pigmented glia (ALSP) and its dysfunction has also been implicated in other neurodegenerative disorders including Alzheimer’s disease (AD). Here, we review the physiological functions of CSF1R in the CNS and its pathological effects in neurological disorders including ALSP, AD, frontotemporal dementia and multiple sclerosis. Understanding the pathophysiology of CSF1R is critical for developing targeted therapies for related neurological diseases.

## Introduction

Microglia are resident innate immune cells in the brain that play crucial roles in the development and homeostasis of the central nervous system (CNS). Colony-stimulating factor 1 receptor (CSF1R) is a product of the proto-oncogene *c-fms* and belongs to the class III transmembrane tyrosine kinase receptor family. CSF1R has been reported to be the major regulator of microglial development and maintenance in the brain (Chitu et al., 2016, 2020). Using small molecule inhibitors targeting CSF1R to eliminate microglia has been implicated in modulating the pathological processes of several neurodegenerative disorders, such as Alzheimer’s disease (AD), Parkinson’s disease (PD), multiple sclerosis (MS), frontotemporal dementia (FTD), and amyotrophic lateral sclerosis (ALS). Although CSF1R is associated with different malignant diseases (Cassier et al., 2015; Cannarile et al., 2017), mutations in CSF1R result in a neurodegenerative disease named adult-onset leukoencephalopathy with axonal spheroids and pigmented glia (ALSP), which is characterized by executive dysfunction, memory decline, personality changes, motor impairments, and seizures (Chitu et al., 2021).

In this review, we provide an overview of the latest findings and implications regarding the role of CSF1R in the CNS. We describe the contribution of CSF1R in microglial population dynamics and neurogenesis, present an overview of genetic CSF1R variants in leukoencephalopathy, and address the pathophysiological function of microglial CSF1R in various neurodegenerative diseases. The cleavage of CSF1R and the potential significance of CSF1R as a target for therapeutic intervention and as a novel diagnostic and prognostic biomarker of disease are also discussed in this article.

## Physiological Role of CSF1R in the Central Nervous System

Microglia, derived from the yolk sac at embryonic day 8.5 (Ginhoux et al., 2010), are broadly distributed throughout the CNS parenchyma. Recent studies reveal remarkably constant and fast turnover of microglia by proliferation and apoptosis by which the number of microglia remains steady from late postnatal stages until aging, suggesting that the whole population in rodents and humans is renewed during a lifetime (Askew et al., 2017). CSF1R is expressed in microglia (including their precursors) (Chitu et al., 2015, 2016; Chitu and Stanley, 2017), neural progenitor cells (NPCs) (Nandi et al., 2012), and several subpopulations of neurons in the CNS (Wang et al., 1999; Nandi et al., 2012; Luo et al., 2013). CNS capillary endothelial cells also express CSF1R (Jin et al., 2014). Homozygous *Csf1r* knockout (*Csf1r*^–/–^) mice exhibit global defects in brain development, including atrophy of the olfactory bulb, expansion of the lateral ventricle, thinning of the neocortex, and functional abnormalities of the sensory nervous system (Erblich et al., 2011; Chitu et al., 2016), reflecting crucial roles of CSF1R in brain development.

### CSF1R and Microglial Maintenance

CSF1R is critical for the development and survival of microglia (Erblich et al., 2011; Chitu and Stanley, 2017). Microglia in the *Csf1r*^–/–^ mouse brain are almost completely depleted at embryonic day 16 and postnatal day 1 (∼99% depletion), and have reduced by ∼94% at 3 weeks of age (Erblich et al., 2011). CSF1R deficiency in rats results in a global loss of resident macrophages, including microglia in the brain (Keshvari et al., 2021). Homozygous CSF1R mutations in the human brain lead to almost complete absence of microglia (Oosterhof et al., 2019). Using an inducible *Cx3cr1* ^CreERT2/+^;*Csf1r*^fl/+^ system to conditional delete one *Csf1r* allele also affects microglial homeostasis (Arreola et al., 2021). CSF1R is highly conserved among vertebrates, including zebrafish, which possess two paralogous genes: *csf1ra* and *csf1rb*. *Csf1rb* was identified to regulate microglia density and distribution, but not microglia differentiation in zebrafish (Oosterhof et al., 2018). However, recently, Ferrero et al. (2021) have uncovered non-overlapping roles for *csf1ra* and *csf1rb* in hematopoiesis, and identified *csf1rb* as an essential regulator of adult microglia development in zebrafish. Altogether, these results emphasize the pivotal role of CSF1R in the maintenance of microglia in vertebrates.

CSF1R is a transmembrane receptor with the tyrosine kinase activity. Two CSF1R ligands, colony stimulating factor 1 (CSF1) and interleukin 34 (IL34), are differentially expressed in the developmental and adult stages in the brain (Droin and Solary, 2010; Nandi et al., 2012). Binding of the ligand, CSF1 or IL34, induces the homodimerization of CSF1R and the activation of downstream signaling pathways (Stanley and Chitu, 2014). CSF1 is expressed in the developing cortex and localized to layer VI of neocortical neurons in the postnatal brain (Nandi et al., 2012). Osteopetrotic (op/op) mice carrying an inactivating mutation in *Csf1* gene exhibit drastic reduction of microglial density in cerebral cortex, corpus callosum and the spinal dorsal column compared to that of wild type animals (Kondo and Duncan, 2009). IL34 is predominantly expressed in the cortex, the anterior olfactory nucleus, and the hippocampus, whereas no or very low expression could be detected in the brain stem and cerebellum (Greter et al., 2012). Using IL34-deficient (*Il34*^LacZ/LacZ^) reporter mice, one study has shown that neurons are the main sources of IL34 in the brain; and IL34 specifically directs the differentiation of microglia in the developing CNS (Wang et al., 2012). CSF1 and IL34 regulate microglial population in a brain region-specific manner (Ginhoux et al., 2010; Greter et al., 2012; Wang et al., 2012; Zelante and Ricciardi-Castagnoli, 2012; Chitu et al., 2016). Functional blocking CSF1 and IL34 in adult mice can deplete microglia differentially in white and gray matter regions of the brain, respectively (Easley-Neal et al., 2019). These results clarify the differential roles of CSF1 and IL34 in microglia development and maintenance in brain regions. Microglia are dramatically reduced by ∼30% in *Csf1*-null brains (Ginhoux et al., 2010) and by ∼70% in adult *Il34*^LacZ/LacZ^ (*Il34*-null) mouse brains (Greter et al., 2012). A recent study has shown that IL34/CSF1R pathway regulates the migration and colonization of microglial precursors in early zebrafish development (Wu et al., 2018).

Interestingly, colony stimulating factor 2 (CSF2), a microglial mitogen, has been found to be elevated in ALSP patients (Kempthorne et al., 2020). *Csf2* heterozygosity prevents cerebral microgliosis of *Csf1r*^+/–^ mice, and microglial homeostasis requires balanced CSF1/CSF2 receptor signaling (Chitu et al., 2020). Notably, CSF1R is a member of the receptor tyrosine kinase family, which can be blocked by kinase inhibitors. Pharmacological studies have also shown that blockade of CSF1R signaling by CSF1R kinase inhibitor, PLX3397, led to an elimination of ∼99% of all microglia brain-wide (Elmore et al., 2014). Interestingly, withdrawal of CSF1R inhibitors leads to a complete repopulation of microglia in the CNS, although treatment with the CSF1R inhibitor is not specific for CSF1R (Waisman et al., 2015; Rice et al., 2017). CSF1R and its ligands (CSF1 and IL34) also control microglial population dynamics during the neuroinflammatory response in the pathophysiology of AD (Martin-Estebane and Gomez-Nicola, 2020). Recently, a study has found that human IL34 drives hematopoietic stem progenitor cell (HSPC)-derived peripheral macrophages into microglial-like cells (Mathews et al., 2019). These results indicate that signaling mediated by CSF1R and its ligands CSF1/IL34 is critical for microglia development and maintenance.

**FIGURE 1:**
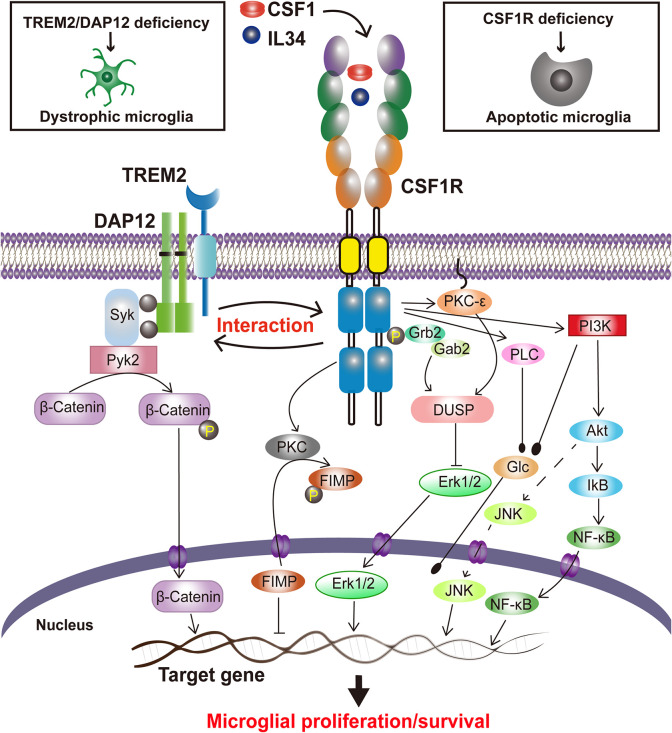
Proposed CSF1R-mediated signaling in microglial survival and proliferation. Stimulated by the ligands, CSF1 or IL34, CSF1Rs are undergoing a rapid dimerization, a self-tyrosine phosphorylation, and activation of PI3K/Akt, JNK, and ERK1/2 pathways, regulating microglia proliferation and survival. CSF1R also interacts with TREM2/DAP12, mediating microglial survival.

CSF1R signaling in cell survival and proliferation has been primarily studied in macrophages. Activated by CSF1 or IL34, CSF1R quickly dimerizes, is auto-phosphorylated, and binds with Grb2/Sos and Cbl, which in turn stimulate macrophage proliferation through activation of the regulatory subunit of PI-3 kinase (PI3K) (p85)/Akt, JNK, and ERK1/2 pathways (Stanley and Chitu, 2014). The PI3K/Akt, phospholipase C (PLC), protein kinase C (PKC), dual specificity phosphatase-1 (DUSP-1) and Fms-interacting protein (FIMP) pathways have been reported to play a role in CSF1R-mediated macrophage survival and/or proliferation (review in Stanley and Chitu, 2014). However, whether similar signaling pathways occur in CSF1R-mediated microglial survival and proliferation (Figure 1) still lacks direct experimental evidence.

Interestingly, our recent study has revealed that Triggering Receptor Expressed on Myeloid cells 2 (TREM2), a microglial innate immune receptor that has been linked to increased AD risks, can interact with CSF1R, which surprisingly is not necessary for CSF1/CSF1R-mediated microglial survival (Cheng et al., 2021). We have also found that CSF1R deficiency significantly reduces the survival of microglia and increases the *Trem2* mRNA level (Cheng et al., 2021). TREM2, however, has also been shown to modulate the proliferation and survival of myeloid cells including microglia (Gratuze et al., 2018; Magno et al., 2021). TREM2 deficiency in primary microglia results in cell cycle arrest at the G1/S checkpoint (Zheng et al., 2017). Indeed, a decrease of microglial proliferation and survival has also been observed when TREM2 expression was reduced (Wang et al., 2015, 2016; Zheng et al., 2017). A recent study has revealed that a putative loss of function *Trem2* Y38C variant alters microglial morphology and number (Jadhav et al., 2020). Additionally, DAP12 (DNAX activating protein of 12 kDa), a transmembrane adaptor of diverse immunoreceptors including TREM2, is activated by CSF1 and mediates the proliferative signals of the CSF1R in myeloid cells (Zou et al., 2008; Otero et al., 2009; Natunen et al., 2020). DAP12 also facilitates the ability of CSF1R to induce the stabilization and nuclear translocation of β-catenin in innate immune cells (McVicar and Trinchieri, 2009) while TREM2 promotes microglial survival by activating the Wnt/β-catenin signaling pathway (Zheng et al., 2017). Interestingly, CSF1R is necessary for microglia viability (Elmore et al., 2014) whereas TREM2 synergizes with CSF1R signaling to sustain microglial survival (Wang et al., 2015). These results suggest a functional trio of CSF1R, TREM2 and DAP12, in which CSF1R plays a crucial role in microglial population dynamics whereas TREM2 and DAP12 are synergistic to the effect (Figure 1). Given that these molecules are specifically expressed in microglia and mutations in either TREM2, DAP12 or CSF1R are associated with neurodegenerative disorders (Klunemann et al., 2005; Rademakers et al., 2011; Chitu et al., 2021), “microgliopathy” is proposed as a new term to designate conditions where microglial dysfunction is the primary and at the center of the disease process (Sasaki, 2017; Zheng et al., 2018).

### Role of CSF1R in Neurogenesis and Neuronal Survival

**FIGURE 2:**
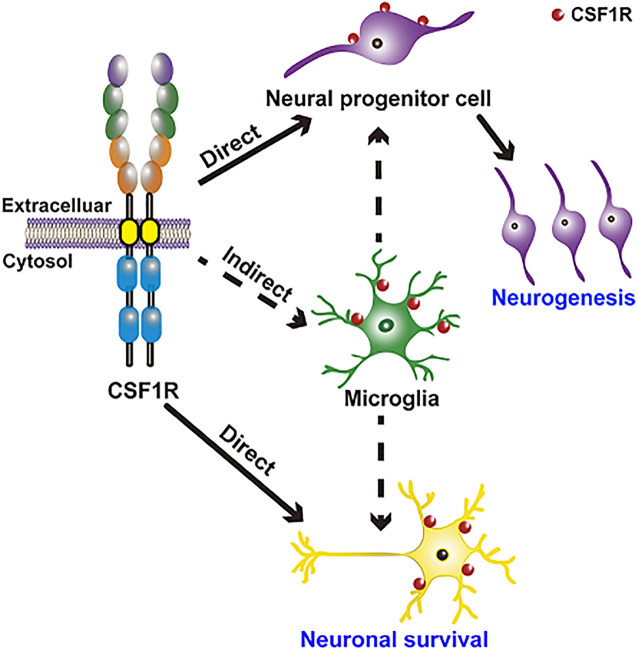
CSF1R regulation of neurogenesis and neuronal survival is achieved in two ways: Direct modulation of NPCs and neurons and indirect modulation through microglia. CSF1R is expressed in NPCs and in a small number of neurons, directly regulating neurogenesis and neuronal survival. CSF1R is also abundantly expressed in microglia, by which can indirectly modulate neurogenesis and the function of neuron.

CSF1R regulation of neurogenesis and neuronal survival is achieved in two ways: direct modulation (embryo) and indirect modulation via microglia (Figure 2). CSF1R is expressed in NPCs (Chitu et al., 2016). CSF1R and its ligands CSF1 and IL34 are shown to regulate the self-renewal, differentiation, and survival of NPCs (Nandi et al., 2012). There are enlarged ventricles and regionally compressed parenchyma with an increased neuronal density in the cortex during the postnatal period of *Csf1r* null mouse (Dai et al., 2002; Erblich et al., 2011; Chitu et al., 2021). Specific deletion of *Csf1r* in NPCs using *Nestin^*Cre/*+^*; *Csf1r*^*fl/fl*^ mice leads to several neurological defects including a smaller brain size, expansion of forebrain neural progenitors and elevated forebrain apoptosis as well as the perinatal lethality, which are presented in the *Csf1r^–/–^* mice (Nandi et al., 2012; Chitu and Stanley, 2017). Using lineage-tracing experiments, Luo et al. (2013) discovered that CSF1R is expressed in a small number of neurons in the hippocampus and cortex under physiological conditions, and its expression is upregulated upon kainic acid-induced excitotoxic injury. Meanwhile, CSF1 and IL34 provide powerful neuroprotective and survival signals in CSF1R-expressed neurons (Luo et al., 2013). These results demonstrate that CSF1R directly regulates neurogenesis and neuronal survival, though the detailed mechanisms need to be clarified in future studies.

Emerging researches indicate that the CSF1R-targeted microglial modulation contributes significantly to the development and maintenance of neurons and neural networks, as well as neurogenesis. CSF1/CSF1R signaling is one of the pathways that are implicated in microglia-mediated neuronal remodeling (Bollinger and Wohleb, 2019). Microglia regulate normal embryonic neurogenesis by phagocytosis of apoptotic cells and by secreting cytokines and chemokines (Thion and Garel, 2017; Shigemoto-Mogami and Sato, 2021). Selective inhibition of microglial proliferation by a CSF1R tyrosine kinase inhibitor (GW2580) reduces neurogenesis and restores normal neuronal differentiation (De Lucia et al., 2016). However, another study has reported that microglia ablation by CSF1R antagonist PLX5622 doesn’t affect the number of neural stem cells (NSCs), transient amplifying cells, and neuroblasts in the subventricular zone (SVZ) niche (Kyle et al., 2019), arguing that microglia are not required for normal functioning SVZ adult neurogenesis. In brief, these results indicate that CSF1R may have complicated roles in neurodevelopmental processes and normal adult neurogenesis.

Elimination of microglia through CSF1R inhibition has also been reported to regulate neural circuit connectivity in adult brain. Microglial depletion via CSF1R inhibition dramatically increases synaptic connections to excitatory cortical neurons as well as the densities and intensities of perineuronal nets (Liu et al., 2021). Long-term microglial depletion via administration of CSF1R inhibitors dramatically increases the dendritic spines in the adult brain (Rice et al., 2015), whereas short-term microglial elimination followed by microglial repopulation increases PSD95 and synaptophysin puncta and promotes functional recovery in mice (Rice et al., 2017). Treating pregnant mice with the CSF1R inhibitor PLX5622 results in approximately 99% microglia elimination from the fetal brain and a decrease in the number of pro-opiomelanocortin (POMC) neurons in the surviving pups (Rosin et al., 2018). These results suggest that targeting CSF1R to eliminate microglia may be beneficial to the treatment against neurological disorders.

### CSF1R Cleavage and Its Biological and Clinical Significance

Regulated intramembrane proteolysis (RIP), which influences diverse cellular processes including differentiation and metabolism, has been observed as a highly conserved process in cells from bacteria to humans (Brown et al., 2000; Murphy et al., 2008). The integral membrane protein first sheds off the extracellular domain of the protein. Then, the truncated membrane-anchored C-terminal fragment (CTF) is cleaved by a second protease, often γ-secretase, within the transmembrane region, resulting in the release of the intracellular domain (ICD), which often translocates from the cytosol into the nucleus to regulate gene transcription (Fortini, 2002).

Several membrane-bound proteins involved in neurodegeneration, including amyloid precursor protein (APP) (Zhang et al., 2011) and TREM2 (Wunderlich et al., 2013; Yang et al., 2020), are proteolytically cleaved to generate a soluble ectodomain and an intracellular protein fragment ICD. The amyloidogenic processing of APP involves sequential cleavage of APP by beta- and gamma-secretases (Xu, 2009; Zhang et al., 2012; Serneels et al., 2020) to give rise to β-amyloid peptides (Aβ) (including Aβ40 and Aβ42) (Hardy and Higgins, 1992; Bai et al., 2021). While a portion of these peptides may form insoluble amyloid deposits in the brain during aging, soluble portion of Aβ40 and Aβ42 are mobilized from the brain interstitial fluid into the cerebrospinal fluid (CSF) and blood; and their concentrations in biofluid are promising diagnostic parameters for AD (Zetterberg and Blennow, 2021). The ICD of APP is released into the cytoplasm and can translocate into the nucleus to regulate gene expression (Zhang and Xu, 2007; Zhang et al., 2007). Similarly, TREM2 undergoes a sequential proteolytic processing by a protease in a disintegrin and metalloproteinase domain (ADAM) family, leading to the shedding of the ectodomain as a soluble TREM2 (Wunderlich et al., 2013). Soluble TREM2 (sTREM2) has been demonstrated to promote microglial survival (Zhong et al., 2017). Furthermore, studies have shown that variants in the extracellular domain of TREM2 results in abnormal shedding (Thornton et al., 2017). Moreover, sTREM2 can also be produced from a transmembrane-domain-trunked TREM2 transcript, which may account for around 25% of the sTREM2 protein levels in the brain cortex (Del-Aguila et al., 2019). However, the role of TREM2 ICD has not been reported.

**FIGURE 3:**
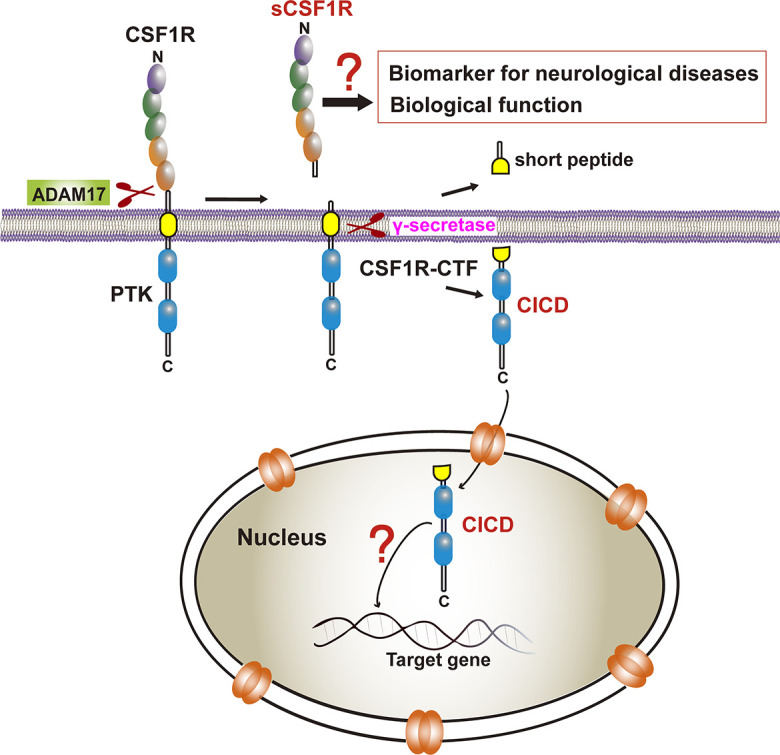
CSF1R cleavage and its biological and clinical significance. CSF1R is sequentially cleaved by ADAM17 and γ-secretase to the release of sCSF1R and CICD. The former may be a biomarker of neurodegenerative diseases although the biological function needs to be determined. The latter translocates into the nucleus yet the transcriptional profiles in microglia remain elusive.

Our recent study also demonstrated that, similar to those of APP and TREM2, a CSF1R ectodomain and a subsequent CSF1R intramembrane can be shed by an ADAM family protease and by gamma-secretase, respectively (Figure 3; Wei et al., 2021). The 972 amino-acid (aa) human CSF1R consists of a 512 aa extracellular ligand-binding domain, a single 25 aa transmembrane segment, and a 435 aa intracellular kinase domain (Coussens et al., 1986). The CSF1R, one of the receptor tyrosine kinases, has also been shown to continuously undergo regulated intramembrane proteolysis (Rovida et al., 2001). ADAM17-deficient hematopoietic progenitors display elevated cell surface CSF1R expression (Becker et al., 2015). CSF1R was identified as a major substrate of ADAM17/TNF-alpha converting enzyme (TACE) in bone marrow progenitors (Qing et al., 2016). Interestingly, the proteolytic shedding of the CSF1R ectodomain can be accelerated by CSF1, granulocyte-macrophage-CSF (GM-CSF), interleukin-2, interleukin-4, and lipopolysaccharide, or by activators of protein kinase C (PKC), such as the phorbol ester 12-O-tetradecanoylphorbol-13-acetate (TPA) or phorbol 12-myristate 13-acetate (PMA) (Downing et al., 1989; Baccarini et al., 1992; Dello Sbarba et al., 1996a,b; Rovida et al., 2001; Wilhelmsen and van der Geer, 2004; Glenn and van der Geer, 2007; Chen and Hung, 2015). Toll-like receptor-induced ERK activation also promotes CSF1R cleavage (Glenn and van der Geer, 2008). The sequence within the extracellular juxtamembrane region is critical for ADAM17/TACE-mediated shedding whereas the transmembrane domain of CSF1R is critical for γ-secretase cleavage. Although Vahidi et al. (2014) have reported TACE and gamma-secretase cleavage sites of CSF1R based on *in vitro* assays in HEK293 cells, the exact cleavage site in human CSF1R has not been identified.

Several studies showed that ligand-induced receptor degradation requires the intrinsic tyrosine kinase activity of CSF1R whereas transmodulation of CSF1R by PKC activator, TPA, the soluble diacylglycerol diC8 or platelet-derived growth factor (PDGF) induced specific proteolytic cleavage of the receptor near its transmembrane segment, resulting in the release of a 100 kDa extracellular ligand-binding domain from the cell surface and the generation of a 50 kDa intracellular fragment containing the kinase domain (Downing et al., 1989; Wilhelmsen and van der Geer, 2004; Vahidi et al., 2014). Additionally, telomerase reverse transcriptase also plays a role in the cleavage of the intracellular domain of CSF1R in epithelial cells resulting a 52 kDa and a 108 kDa fragments (Li et al., 2009). Interestingly, a soluble form of CSF1R (sCSF1R) was reported in goldfish serum (Barreda et al., 2005). Moreover, a peptide derived from CSF1R has been identified in human cerebrospinal fluid (CSF) which can provide an area under curve (AUC) of 0.873 (sensitivity = 76.7%, specificity = 80.0%) as a potential biomarker for PD vs. healthy controls (Shi et al., 2015). Given that Aβ40/Aβ42 from APP cleavage in CSF and blood have been reported to correlate with brain pathology (Dey et al., 2019; Wang H. et al., 2020) and that soluble TREM2 in human cerebrospinal fluid has been identified to be a reliable predictor of the early stages of AD (Piccio et al., 2008; Ewers et al., 2019; Suarez-Calvet et al., 2019; Ma et al., 2020; Yang et al., 2020), whether sCSF1R can be determined in human cerebrospinal fluid or the serum of neurodegenerative patients as a biomarker awaits to be explored (Figure 3). Furthermore, higher CSF sTREM2 attenuates ApoE4-related risk for cognitive decline and neurodegeneration in AD, which provides evidence that sTREM2 may be protective against the development of AD (Franzmeier et al., 2020). Thus, it’s attractive to clarify the biological function of sCSF1R in CNS in future work.

**FIGURE 4:**
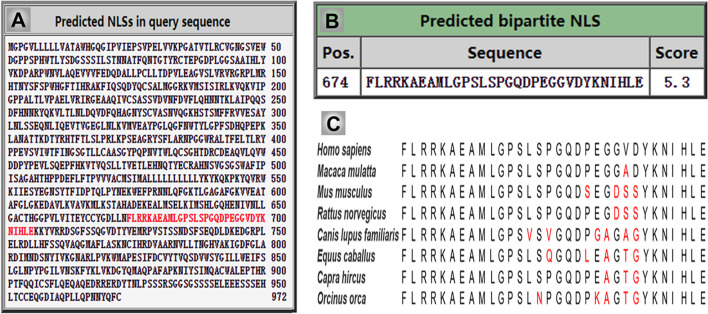
The intercellular domain of CSF1R (CICD) contains nuclear localization signal (NLS), which is highly conserved in vertebrates. **(A)** The NLS sequence of human CSF1R is predicated and indicated in red using cNLS Mapper. **(B)** The score of human CSF1R NLS sequence is 5.3, which indicates the high possibility of its role in translocating CICD into the nucleus. **(C)** Homologous comparison of the NLS sequence of CSF1R in different vertebrate species.

Importantly, previous studies indicated that the CSF1R may be localized within the nucleus (Glenn and van der Geer, 2007, 2008; Rovida and Dello Sbarba, 2014). CSF1R is present in nuclear extracts of the epithelial cells by immunoblot analysis of the cell component extracts (Li et al., 2009). Interestingly, one study has found that the full-length CSF1R migrates to the nucleus upon CSF1 stimulation in human primary monocytes. When monocytes are differentiated into macrophages in response to CSF1, CSF1R is found to interact with H3K4me3 and several transcription factors, such as Eag-like K[^+^] channel (ELK) and yin yang 1 (YY1) (Bencheikh et al., 2019), indicating a dynamic role of nuclear CSF1R in the monocytic lineage. In fact, based on the program^1^ made by Kosugi et al. (2009) the ICD of CSF1R (CICD) contains a putative nuclear localization signal (NLS), which is highly conserved in vertebrates (Figure 4) (Kosugi et al., 2009). Further studies are needed to clarify the specific role of nuclear CICD in the transcriptional profiles in those cells (Figure 3).

## CSF1R in Neurodegenerative Diseases

### CSF1R Mutations Are the Major Cause of Adult-Onset Leukoencephalopathy With Axonal Spheroids and Pigmented Glia

CSF1R signaling is important for the homeostatic, survival, proliferation and differentiation of the macrophage lineage cells, including microglia. Mutations within the *CSF1R* gene result in a subtype of dominantly inherited leukoencephalopathy named adult-onset leukoencephalopathy with axonal spheroids and pigmented glia (ALSP), which is regarded as a single clinicopathologic entity of hereditary diffuse leukoencephalopathy with spheroids (HDLS) and pigmentary orthochromatic leukodystrophy (POLD) (Wider et al., 2009; Rademakers et al., 2011; Nicholson et al., 2013; Konno et al., 2014; Wider and Wszolek, 2014). ALSP typically develops in adulthood, with cognitive decline, psychiatric symptoms, and movement disorders of gait disturbance and bradykinesia. The pathology of ALSP is characterized by giant neuroaxonal swellings (spheroids) within the CNS white matter.

The symptoms of ALSP are variable. Konno et al. (2018b) have established and validated diagnostic criteria for ALSP to specifically differentiate it from other leukoencephalopathies. However, there are no biomarkers for the diagnosis of ALSP. The levels of neurofilament light chain (NfL), a polypeptide important for the structural integrity of axons, in both serum and CSF have been found to be considerably higher in patients with ALSP (Hayer et al., 2018a). Given that NfL has been utilized as a diagnostic and prognostic biomarker of several neurodegenerative diseases (Ng et al., 2020; Zucchi et al., 2020; Barth et al., 2021; Fagan et al., 2021; Meca-Lallana et al., 2021; Pekny et al., 2021; Witzel et al., 2021), it may also be a potential biomarker for ALSP. Notably, a recent study reveals that diffusion-weighted imaging may facilitate decisive diagnosis of specific leukoencephalopathy, including ALSP (Tokumaru et al., 2021), whereas another study emphasized the great variability of MRI findings seen in ALSP (Codjia et al., 2018). Due to the heterogeneous clinical presentations, HDLS patients are often misdiagnosed with other diseases. Early in 2013, Sundal et al. (2013) analyzed sixteen CSF1R mutation carriers, none of whom had been given an initial clinical diagnosis of HDLS, and found atypical Parkinsonian features in those HDLS. Actually, using the NeuroChip, Blauwendraat et al. (2019) analyzed rare variants of damaging mutations in 1243 autopsy-confirmed neurodegenerative cases that were diagnosed as AD, FTD or ALS in the Johns Hopkins Brain Resource Center, and identified 2 cases with a pathogenic CSF1R mutation (p.G589E, and p.M755T or M766T) and 1 case with a likely pathogenic CSF1R mutation (p.L868P). Thus, there are complex genotype-phenotype associated with ALSP (Guo and Ikegawa, 2021).

**FIGURE 5:**
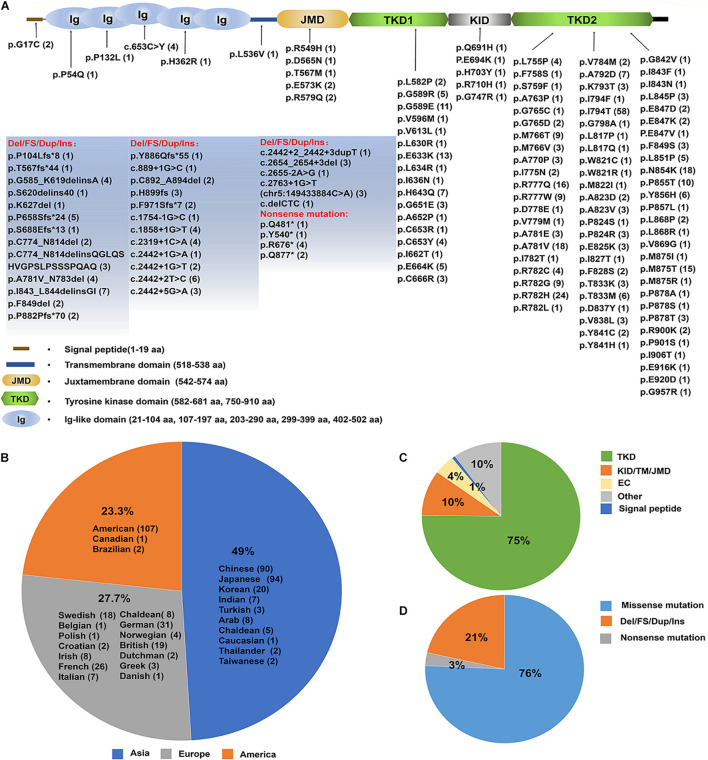
An update on the spectrum of CSF1R mutations causing ALSP. **(A)** A diagram of CSF1Rprotein structure. The brown line represents N-terminal signal peptide of CSF1R. Ig1–Ig5 ovals represent the five extracellular Ig-like domains of CSF1R. The transmembrane domain is illustrated in light blue. The intracellular domain is shown as the juxtamembrane domain (JMD, light yellow), tyrosine kinase domain (TKD, light green), kinase insert domain (KID, light gray) and carboxy-terminal tail (black). The TKD of CSF1R is interrupted by the KID. Up to date, a total of 140 pathogenic mutations have been identified (indicated and listed below). The numbers in the parentheses represent the number of reported cases. **(B)** Emerging cases have been reported in the United States, Europe and Asia. The number of cases in each region is listed in the parentheses. **(C)** A pie-chart illustrates the mutation locations for a total of 140 pathogenic CSF1R variants. 64% mutations are located within TKD, 4% within the extracellular domain (ED), and 8% within Juxtamembrane domain (JMD), KID or Transmembrane domain (TM). **(D)** A pie-chart illustrates the type of mutations for a total of 464 cases with pathogenic CSF1R variants identified up to date, which include point mutations, nonsense mutations, frameshift and deletion mutations. The majority of the CSF1R variants are point mutations. NA, not available.

Nonetheless, the number of ALSP patients confirmed by genetic diagnosis of CSF1R has been increasing in various populations. ALSP has an expanding mutational spectrum and phenotypic presentation and within the last decade, mutations in the CSF1R have been shown to cause rare diseases of both pediatric and adult onset (Chitu et al., 2021). Up to date, 473 cases with CSF1R mutations were identified (see details in Supplementary Table 1), of which 49% located in Asia, 23.3% in United States and 27.7% in Europe (Figures 5A,B and Supplementary Table 2). More than 140 pathogenic CSF1R variants have been reported in those patients with ALSP. Most of the mutations (roughly 75%) are located in the tyrosine kinase domain (TKD) of CSF1R resulting in impaired kinase activity and downstream signaling of CSF1R (Figures 5A,C and Supplementary Table 3). 76% of the variants are missense mutations, 18% are deletions/frameshift/duplicate/insert (Del/FS/Dup/Ins) and 2% nonsense mutations (Figures 5A,D).

Notably, bi-allelic and homozygous CSF1R mutations have also been recently reported in CSF1R-related leukoencephalopathy (Guo et al., 2019; Oosterhof et al., 2019; Tamhankar et al., 2020; Kindis et al., 2021). However, one study reported two unrelated individuals carrying homozygous CSF1R mutations with disease presentation distinct from ALSP (Oosterhof et al., 2019). Additionally, others have reported that CSF1R mutation carriers are clinically unaffected, indicating the possible incomplete penetrance of these CSF1R mutations (Karle et al., 2013; Abe et al., 2017; Tipton et al., 2021).

Emerging studies have also identified recessive mutations in *alanyl-transfer (t)RNA synthetase 2 (AARS2)* gene, which encodes a tRNA synthetase responsible for correctly loading alanine onto tRNA-ala for the translation of mitochondrial proteins. AARS2 mutation carriers developed ALSP with an average age of onset around 29 years of age (range 15–44 years) (Lynch et al., 2016, 2017, 2019; Taglia et al., 2018; Wang et al., 2018; Axelsen et al., 2021). It is intriguing why these two seemingly unrelated genes, *CSF1R* and *AARS2*, give rise to the same leukodystrophy. Given that AARS2 is responsible for the translation of mitochondrial proteins and that mitochondria dysfunction is essential in the pathogenesis of neurological diseases such as AD (Wang W. et al., 2020), it is possible that both CSF1R and AARS2 deficiency affects the mitochondrial structural and functional integrity. Future investigations on potential functional correlations between CSF1R and AARS2 may shed light on whether and how CSF1R and AARS2 mutations lead to leukodystrophy through common molecular mechanisms.

Although CSF1R mutations cannot account for all the ALSP cases (one ALSP case has been reported with patients carrying no known variants associated with the disease (Fernandez-Vega et al., 2016)), it is still the most predominant cause of ALSP. Understanding CSF1R-related disease-causing mechanisms may facilitate the discovery and development of effective therapies.

### Possible Mechanisms and Promising Treatments of Adult-Onset Leukoencephalopathy With Axonal Spheroids and Pigmented Glia Targeting CSF1R

ALSP is a form of dementia resulting from dominantly inherited CSF1R inactivating mutations. Fewer and dysmorphic microglia as well as an inflammatory phenotype of microglia were found in the brains of ALSP patients (Tada et al., 2016; Kempthorne et al., 2020). The *Csf1r*^+/–^ mouse is sufficient to mimic ALSP symptoms and pathology (Chitu et al., 2015). Given that CSF1R mainly expresses in microglia, CSF1R-related leukoencephalopathy is representative of primary microgliopathies, of which microglia have a pivotal and primary role in pathogenesis (Konno et al., 2018a; Kempthorne et al., 2020). Indeed, using *Cx3cr1*^Cre/+^;*Csf1r*^fl/+^ mice with heterozygous deletion of mouse *Csf1r* in microglia, a recent study has demonstrated that ALSP is a primary microgliopathy (Biundo et al., 2021). Studies have also revealed that *Csf1r* heterozygosity results in enhanced cerebral Csf2, CD68 and CD163 expression, indicating an inflammatory phenotype of microglia in affected white matter of ALSP (Chitu et al., 2015; Kempthorne et al., 2020). Intriguingly, monoallelic deletion of Csf2 in the *Csf1r*^+/–^ mouse model of ALSP rescues most behavioral deficits and histopathological changes in those mice (Chitu et al., 2020). Additionally, axonal swelling is a characteristic neuropathology of ALSP caused by CSF1R mutation. Dystrophic axons near amyloid plaque, known as dystrophic neurites (DNs), is also a major and distinguishing pathology of AD that has a sequential organization with numerous proteins/organelles surrounding amyloid core (Sharoar et al., 2019, 2021). Hence, potential effects of CSF1R or CSF1R-mediated microglia depletion on DNs formation may also play important role in neuropathology of ALSP and AD. CSF1R inhibitor PLX5622 prevented the loss of presynaptic surrogates and the extracellular matrix (ECM) structure perineuronal nets in *Csf1r*^+/–^ mice (Arreola et al., 2021). An induced pluripotent stem cell line from a patient with ALSP has been generated, which may allow the investigation of CSF1R mutations in a human cell model and shed new light on the therapeutic strategies of this intractable ALSP (Hayer et al., 2018b). Moreover, recent studies have developed efficient strategies for microglia replacement by bone marrow transplantation, peripheral blood, or microglia transplantation, which may potentially open up new possibilities for treating microglia-associated CNS disorders (Speicher et al., 2019; Xu et al., 2020). Actually, hematopoietic stem cell transplantation (HSCT) has been reported to achieve beneficial effectiveness in patients with ALSP, indicating that homeostatic microglia replacement is a promising approach in ALSP treatment (Mochel et al., 2019; Gelfand et al., 2020; Han et al., 2020). Genetic models and rudimentary pharmacologic approaches for microglial depletion and/or repopulation of microglia have been investigated for the therapeutic strategies in neurodegenerative disorders, especially Alzheimer’s disease (Graykowski and Cudaback, 2021). Studies revealed that pharmacological CSF1R inhibitors afford the most extensive versatility in manipulating microglia, making them ideal candidates for future therapy of neurodegenerative disorders (Green et al., 2020; Green and Hume, 2021). These results highlight future therapeutic and experimental approaches of ALSP targeting CSF1R.

### Possible Roles of CSF1R in Alzheimer’s Disease and Other Neurodegenerative Diseases

CSF1R mutation carriers may present clinical phenotypes of AD, FTD, PD, and MS, accompanied with white matter abnormalities.

#### CSF1R in Alzheimer’s Disease

Amyloid-beta plaque, tau neurofibrillary tangle deposition and neuroinflammation are three key pathological features of AD (Hyman et al., 2012; Guo et al., 2020; Chung et al., 2021; Xia et al., 2021). *APP*, *PSEN1*, and *PSEN2* are causative genes for AD (Reitz et al., 2020; Ibanez et al., 2021; Seto et al., 2021). The *APOE ε4* allele and variants of TREM2 are major genetic risk factors for late-onset AD whereas *APOE ε2* decreases the risk of late-onset AD (Kanekiyo et al., 2014; Li et al., 2020; Williams et al., 2020; Shafi et al., 2021). Remarkably, studies provide evidences of an association between CSF1R variants (p.P54Q, p.L536V, p.L868R, p.Q691H, and p.H703Y) and AD risk (Blue et al., 2018; Sassi et al., 2018; Giau et al., 2019). Moreover, CSF1R has also been identified as one of the molecular networks in regulating disease-associated microglia in the pathophysiology of AD (Xu et al., 2021).

Interestingly, CSF1R has been observed to play potential role in disrupted lipid metabolism in *Csf1r knockout* livers (Keshvari et al., 2021) and the corpora callosa of patients with schizophrenia (Shimamoto-Mitsuyama et al., 2021). Meanwhile, TREM2 has also been identified as a microglial lipid-sensor, and recent data indicate lipid droplets accumulation in microglia in AD (Claes et al., 2021). Additionally, *Trem2* deficiency in APP/E4 mice significantly decreased plaque-associated APOE protein (Fitz et al., 2020), which plays an essential role in lipid trafficking, cholesterol homeostasis, and synaptic stability (Liu et al., 2013). In addition, TREM2 mutations and deficiency have been shown to regulate APOE expression at the epigenetic level (Liu T. et al., 2020). These recent findings post additional questions on whether CSF1R affects APOE protein level and lipid metabolism and whether the effects may be in an APOE isoform-dependent manner.

CSF1R signaling has also been reported to be critical in the pathological progress in AD. Increased CSF2 levels and decreased microglial CSF1R expression have been found in AD (Chitu et al., 2020). A study has shown that systemic administration of human recombinant CSF1, one of the CSF1R ligands, ameliorates memory deficits in an AD mouse model (Luo et al., 2013). Zuroff et al. (2020) found that IL34, another ligand of CSF1R, impairs monocyte differentiation into macrophages and reduces their ability to uptake pathological forms of Aβ. Furthermore, one study has shown that cognitive decline is delayed in CSF1R-deleted APPSwe/PS1mice, in which TREM2/ β-catenin and IL34 expression are significantly increased (Pons et al., 2021), indicating that conditional deletion of CSF1R in microglia ameliorates the physiopathology of AD.

Microglia in the brain depend on CSF1R signaling for survival, and pharmacological inhibition of this receptor results in the elimination of nearly all microglia in the CNS (Elmore et al., 2014). Dysfunction of microglia aggravates the pathogenesis of AD. Therefore, targeting CSF1R to eliminate or repopulate microglia in the brain may intervene the onset and/or progression of AD. Accumulative studies have shown microglia-mediated regulation of plaque deposition and/or p-tau propagation by CSF1R inhibitors. For example, the prevention of early microglial proliferation by a CSF1R inhibitor (GW2580) hinders the development of senescence and disease-associated microglia, impairing the accumulation of Aβ, as well as associated neuritic and synaptic damage (Hu et al., 2021). Moreover, CSF1R-inhibition-mediated microglia deletion protects against plaque-dependent perineuronal nets (PNNs) loss in the AD brain (Crapser et al., 2020), prevents plaque deposition, and improves cognition in different AD-related mouse models (Dagher et al., 2015; Olmos-Alonso et al., 2016; Sosna et al., 2018; Spangenberg et al., 2019). However, Spangenberg et al. (2016) have shown chronic microglial elimination does not alter plaque load but protects against dendritic spine loss, neuronal loss, neuroinflammation and memory impairment. A recent study from Benitez et al. (2021) showed that using a CSF1R inhibitor to eliminate microglia had little effect on plaque load in *App* knock-in mice. Studies also revealed that CSF1R inhibitor-induced microglial repopulation in aged mice (24 months) significantly improved the spatial memory to the level comparable to that of young adult animals (4 months) (Elmore et al., 2018); and that PLX5622-elicited microglial repopulation resulted in more compact plaques predominating microglia-repopulated regions (Casali et al., 2020). Additionally, CSF1R has also been reported to play a role in tauopathy. Blockade of microglial proliferation by CSF1R inhibitor JNJ-40346527 (JNJ-527) attenuated tau-induced neurodegeneration in the P301S mouse tauopathy model (Mancuso et al., 2019). Lodder et al. (2021) revealed that pharmacological targeting of CSF1R preferentially eliminated non-plaque-associated microglia and rescued tau pathology and neurodegeneration in a combined AD pathologies mouse model, while preserving protective plaque associated microglia. Administration of PLX5622 dramatically reduced propagation of p-tau in App^NL–*G*–F^ mouse, a humanized APP mutant knock-in homozygote mouse (Clayton et al., 2021). However, one study reported that using PLX3397, another CSF1R inhibitor, to reduce the number of microglia, did not change the tau burden nor astrocyte activation in the brains of a tau-overexpressing Tg4510 mouse model (Bennett et al., 2018), which may account for the 30% reduction of microglial numbers by PLX3397. The effects of the CSF1R inhibitors (such as PLX5622, PLX3397, JNJ-527, and GW2580) on microglia depletion, amyloid/tau pathology and synaptic function are shown in Supplementary Table 4. Collectively, these results demonstrate that CSF1R inhibitors represent potential preventative or therapeutic approach in AD, yet a better understanding of specific microglial changes are required.

#### CSF1R in Other Neurodegenerative Diseases

Several studies have highlighted the role of pathogenic mutations in CSF1R originate in microglia contributing to other neurodegenerative diseases, including FTD (Sirkis et al., 2019, 2021; Lok and Kwok, 2021). One study identified a novel in-frame deletion (c.2675_2683del) in the CSF1R gene in a patient with behavioral variant FTD (bvFTD) who possessed severe bifrontal atrophy with frontal subcortical white matter changes (Kim et al., 2018). Another case has shown a bvFTD patient harboring a likely pathogenic variant in CSF1R (c.2699G > A, p.Arg900Lys) (Cochran et al., 2019). Sassi et al. (2021) performed whole exome sequencing in 100 patients with a late-onset and heterogeneous FTD-like clinical phenotype and identified two sporadic cases with behavioral FTD carring heterozygous CSF1R p.E573K and p.R549H mutations. Sharma et al. (2019) reported an HDLS patient carrying a known pathogenic variant [2381T > C (p.Iso794Thr)] in the *CSF1R* gene but presenting clinical symptoms and brain imaging features similar to that of Dementia with Lewy Bodies (DLB).

One study assessed the potential association of two functional genetic variants, namely CSF1 rs1058885 and CSFR1 rs10079250, in a cohort including 502 Taiwanese PD patients and 511 age- and gender-matched healthy controls. While CSF1R rs10079250 polymorphism is not associated with the risk of PD in this cohort, CSF1 rs1058885 TT variant appears to be less frequent in PD patients than in control subjects (Chang et al., 2019), suggesting that CSF1/CSF1R neuroinflammatory signaling may also be involved in the pathogenesis of PD. Additionally, pharmacological microglial depletion using CSF1R inhibitor GW2580 significantly attenuates the dopamine neuron loss and motor behavioral deficits in a PD-related mouse model (Neal et al., 2020), indicating that targeting CSF1R signaling may be a viable neuroprotective strategy in PD.

MS is a chronic inflammatory disease affecting the CNS. Although previous studies didn’t find CSF1R as a common cause of MS (Sadovnick et al., 2013), recent work from Li et al. identified CSF1R among the eight core genes correlated with MS using raw data of two independent datasets from the ArrayExpress database (Li et al., 2021). Several motor predominant cases of ALSP caused by heterozygous CSF1R mutations has been reported to mimic primary progressive multiple sclerosis (PPMS) (Inui et al., 2013; Saitoh et al., 2013; Sundal et al., 2015; Prieto-Morin et al., 2016).

CSF1R-targeted microglial elimination also plays key roles in MS. CSF1R inhibition by BLZ945 in the 5-week murine cuprizone model enhances central remyelination by modulating neuroinflammation (Beckmann et al., 2018). Treatment with CSF1R inhibitor GW2580 or PLX5622 significantly reduced the severity, and prevented the progression of MS in an experimental allergic encephalomyelitis (EAE) animal model (Borjini et al., 2016; Nissen et al., 2018). Moreover, CSF1R inhibitor PLX3397 greatly abrogates the demyelination, loss of oligodendrocytes, and reactive astrocytosis in the cuprizone model of MS (Tahmasebi et al., 2019; Marzan et al., 2021). CSF1R inhibition can reduce deleterious microglial proliferation and modulate microglial phenotypes during neuroinflammatory pathogenesis, particularly in progressive MS (Hagan et al., 2020). CSF1 was upregulated both in blood, CSF and the spinal cord of MS patients. These findings provide clear evidence that CSF1R signaling is involved in MS.

However, several studies also have shown the disadvantage of microglia deletion in disease processes. Pharmacological ablation of microglia with the CSF1R inhibitor PLX5622 led to aggravated vascular calcification (Zarb et al., 2021), and accelerated prion disease (Carroll et al., 2018). Although one study reported that microglia depletion by PLX3397 exacerbated seizure severity and excitotoxicity-induced neuronal degeneration (Liu M. et al., 2020), other studies predicted CSF1R to be a potential target for anti-epileptic drugs (Lyman and Chetkovich, 2019; Gerard et al., 2021) as CSF1R blockade attenuating epilepsy seizures has been validated in three pre-clinical models of epilepsy (Srivastava et al., 2018). These results highlight the complicated role of CSF1R inhibition meditated microglia in neurological diseases. Hence, differentially alter the role of CSF1R-mediated microglial activation during neurodegenerative disease progression may provide a unique opportunity for more suitable and effective therapeutics.

## Conclusion and Prospections

Altogether, accumulative studies have provided solid evidence of a central role of the CSF1R in the CNS. Given the lack of effective diagnostic criteria for CSF1R associated neurological diseases, the soluble form of CSF1R may be a promising biomarker and therapeutic target for CSF1R-associated neurodegenerative disorders. Moreover, elucidating the biological role of CSF1R ICD will lead to a better understanding of the underlying molecular mechanisms involved in the progress of CSF1R related leukoencephalopathy. The spectrum of neurological diseases associated with CSF1R variants has substantially increased in recent years and is still ongoing, which calls for in-depth mechanistic investigations on CSF1R’s diverse roles in these diseases at the different disease stages. No CSF1R mutation knock-in animal model is currently available. Generation of such models may provide valuable tools to elucidate the underlying CSF1R-related disease mechanisms. Both microglia replacement and CSF1R inhibitor-mediated microglia modulation may be attractive strategies to specifically treat CSF1R-associated neurodegenerative disorders. Thus, microglial CSF1R is highlighted as emerging targets for disease-modifying therapy in ALSP and other neurological disorders.

## Author Contributions

HZ and HX contributed to the conception of the article. HZ performed literature research and drafted the manuscript. BH, SD, and ZW prepared the illustrations. XL and YZ revised the manuscript. Y-WZ and HX reviewed and edited the manuscript before submission. All authors approved the final version.

## Conflict of Interest

The authors declare that the research was conducted in the absence of any commercial or financial relationships that could be construed as a potential conflict of interest.

## Publisher’s Note

All claims expressed in this article are solely those of the authors and do not necessarily represent those of their affiliated organizations, or those of the publisher, the editors and the reviewers. Any product that may be evaluated in this article, or claim that may be made by its manufacturer, is not guaranteed or endorsed by the publisher.

## Footnotes

1
https://nls-mapper.iab.keio.ac.jp/cgi-bin/NLS_Mapper_form.cgi
