# Supplementary material for: Insights Into the Role of CSF1R in the Central Nervous System and Neurological Disorders
Source: Front Aging Neurosci. 2021 Nov 15;13:789834. doi: 10.3389/fnagi.2021.789834 (PMC8634759; doi:10.3389/fnagi.2021.789834)
Supplement: Supplementary file 1 [file Table_1.pdf]

Supplementary Table 1. *CSF1R* mutations in ALSP.

Protein domain	Region	Nationality	Mutation Type	Protein Mutation	Sex	Case	Reference	Notes
Signal peptide	Asia	Chinese	Missense mutation	p.G17C	2 M	2	[1, 2]	
EC	Asia	Thailander	Missense mutation	p.P54Q	1 NA	1	[3]	
EC	North America	American	Del/FS/Dup/Ins	p.P104Lfs*8	1 F	1	[4]	Cause nonsense-mediated RNA decay (NMD)
EC	South America	Brazilian	Missense mutation	p.P132L	1 M	1	[5]	Bi-allelic Mutations
EC	Asia	Japanese	Missense mutation	c.653 C > Y	1 M, 3 F	4	[6]	Carrier: 1 M, 1 F
EC	North America	American	Missense mutation	p.H362R	1 M	1	[4]	Homozygous mutation
EC	South America	Brazilian	Nonsense mutation	p.Q481*	1 M	1	[5]	Bi-allelic Mutations
TM	Asia	Thailander	Missense mutation	p.L536V	1 NA	1	[3]	
JMD	Asia	Arab	Nonsense mutation	p.Y540*	1 NA	1	[7]	
JMD	Europe	Italian	Missense mutation	p.R549H	1 NA	1	[8]	
JMD	Europe	Caucasian	Missense mutation	p.D565N	1 NA	1	[9]	
JMD	North America	American	Del/FS/Dup/Ins	p.T567fs*44	1 F	1	[10]	
JMD	Asia	Chinese	Missense mutation	p.T567M	1 F	1	[11]	
JMD	North America	American	Missense mutation	p.E573K	1 M	1	[12]	
JMD	Europe	Italian	Missense mutation	p.E573K	1 NA	1	[8]	
JMD	Asia	Chinese	Missense mutation	p.R579Q	2 F	2	[1]	
TKD1	Europe	German	Missense mutation	p.L582P	2 M	2	[13, 14]	
TKD1	Europe	Norwegian	Del/FS/Dup/Ins	p.G585_K619delinsA	2 F, 2 NA	4	[15, 16]	

Protein domain	Region	Nationality	Mutation Type	Protein Mutation	Sex	Case	Reference	Notes
TKD1	North America	American	Missense mutation	p.G589E	4 M, 7 F	11	[15-19]	
TKD1	Asia	Caucasian	Missense mutation	p.G589R	1 F	1	[20]	
TKD1	Asia	Japanese	Missense mutation	p.G589R	4 F	4	[19, 21, 22]	
TKD1	Europe	British	Missense mutation	p.V596M	1 F	1	[23]	
TKD1	Asia	Chinese	Missense mutation	p.V613L	1 F	1	[1]	
TKD1	Asia	Japanese	Del/FS/Dup/Ins	p.S620delins40	1 F	1	[5]	Bi-allelic Mutations
TKD1	Asia	Japanese	Del/FS/Dup/Ins	p.K627del	1 F	1	[5]	Bi-allelic Mutations
TKD1	Europe	Dutchman	Missense mutation	p.L630R	1 M	1	[10]	
TKD1	Europe	Greek	Missense mutation	p.E633K	1 F, 1 NA	2	[10, 23]	
TKD1	North America	American	Missense mutation	p.E633K	4 M, 3 F, 2 NA	9	[15, 16]	
TKD1	Europe	Danish	Missense mutation	p.E633K	1 F	1	[24]	
TKD1	Europe	German	Missense mutation	p.E633K	1 F	1	[14]	
TKD1	Europe	British	Missense mutation	p.L634R	1 F	1	[25]	
TKD1	Asia	Chinese	Missense mutation	p.I636N	1 M	1	[26]	
TKD1	Asia	Arab	Missense mutation	p.H643Q	5 M, 2 F	7	[27]	2 homozygous mutations
TKD1	Asia	Chinese	Missense mutation	p.G651E	1 M, 2 F	3	[28, 29]	
TKD1	Asia	Japanese	Missense mutation	p.A652P	1 F	1	[19]	
TKD1	Europe	Italian	Missense mutation	p.C653R	1 F	1	[30]	
TKD1	Asia	Japanese	Missense mutation	p.C653Y	1 M, 3 F	4	[31]	Carrier: 1 M, 2 F
TKD1	Asia	Chaldean	Del/FS/Dup/Ins	p.P658Sfs*24	1 M, 4 F	5	[5]	Bi-allelic Mutations
TKD1	North America	American	Missense mutation	p.I662T	1 M	1	[4]	

Protein domain	Region	Nationality	Mutation Type	Protein Mutation	Sex	Case	Reference	Notes
TKD1	North America	American	Missense mutation	p.E664K	1 M, 4 F	5	[32]	
TKD1	North America	American	Missense mutation	p.C666R	1 M, 2 F	3	[33]	
TKD1	Asia	Chinese	Nonsense mutation	p.R676*	4 F	4	[26, 34]	
KID	Asia	Japanese	Del/FS/Dup/Ins	p.S688Efs*13	1 F	1	[35]	
KID	Europe	Caucasian	Missense mutation	p.Q691H	1 F	1	[9]	
KID	Europe	Caucasian	Missense mutation	p.E694K	1 NA	1	[9]	
KID	Europe	Caucasian	Missense mutation	p.H703Y	1 NA	1	[9]	
KID	North America	American	Missense mutation	p.R710H	1 NA	1	[15]	
KID	North America	American	Missense mutation	p.G747R	1 NA	1	[12]	
TKD2	Asia	Chinese	Missense mutation	p.L755P	2 M, 2F	4	[36]	Carrier: 1 M
TKD2	Asia	Japanese	Missense mutation	p.F758S	1 F	1	[37]	
TKD2	Asia	Japanese	Missense mutation	p.S759F	1 M	1	[38]	
TKD2	Europe	British	Missense mutation	p.A763P	1 M	1	[23]	
TKD2	Asia	Japanese	Missense mutation	p.G765C	1 NA	1	[39]	
TKD2	Asia	Japanese	Missense mutation	p.G765D	1 M,1 F	2	[4, 35]	
TKD2	North America	American	Missense mutation	p.M766T	3 M, 6 F	9	[16, 17, 19, 40]	
TKD2	Asia	Korean	Missense mutation	p.M766V	3 M	3	[41]	
TKD2	Europe	French	Missense mutation	p.A770P	1 M	1	[42]	
TKD2	Europe	German	Missense mutation	p.A770P	1 M, 1NA	2	[15, 16]	
TKD2	North America	American	Del/FS/Dup/Ins	p.C774_N814del	2 M	2	[15, 16]	
TKD2	North America	American	Del/FS/Dup/Ins	p.C774_N814delinsQGLQ SHVGPSLPSSSPQAQ	2 F, 1 NA	3	[15, 16, 19]	

Protein domain	Region	Nationality	Mutation Type	Protein Mutation	Sex	Case	Reference	Notes
TKD2	North America	American	Missense mutation	p.I775N	2 NA	2	[15, 16]	
TKD2	North America	American	Missense mutation	p.R777Q	2 F	2	[28, 43]	
TKD2	Europe	French	Missense mutation	p.R777Q	4 M, 1 F	5	[10, 42]	
TKD2	Europe	German	Missense mutation	p.R777Q	3 M, 2 F	5	[44, 45]	
TKD2	Asia	Japanese	Missense mutation	p.R777Q	1 M, 1 F, 2 NA	4	[39, 46, 47]	
TKD2	Asia	Chinese	Missense mutation	p.R777W	1 M	1	[48]	
TKD2	Europe	British	Missense mutation	p.R777W	1 F	1	[10]	
TKD2	Asia	Japanese	Missense mutation	p.R777W	5 M, 2 NA	7	[31, 38, 39]	
TKD2	North America	American	Missense mutation	p.D778E	1 F	1	[4]	
TKD2	Asia	Japanese	Missense mutation	p.V779M	1 NA	1	[39]	
TKD2	Asia	Japanese	Missense mutation	p.A781E	1 F	1	[35]	
TKD2	Asia	Chinese	Missense mutation	p.A781E	2 M	2	[26]	
TKD2	Europe	British	Missense mutation	p.A781V	4 M, 1 F	5	[49]	
TKD2	Asia	Chinese	Missense mutation	p.A781V	1 M, 1 F	2	[26, 34]	
TKD2	Europe	French	Missense mutation	p.A781V	2 F	2	[42, 50]	
TKD2	Europe	German	Missense mutation	p.A781V	2 M, 1 F	3	[14, 45]	
TKD2	Asia	Korean	Missense mutation	p.A781V	3 M, 3 F	6	[41]	Carrier: 2 M, 1 F
TKD2	Europe	Irish	Del/FS/Dup/Ins	p.A781V_N783del	2 M, 2 F	4	[51]	
TKD2	Asia	Japanese	Missense mutation	p.R782G	6 F	6	[21]	Carrier: 2 F
TKD2	Asia	Japanese	Missense mutation	p.I782T	1 F	1	[52]	
TKD2	Asia	Chinese	Missense mutation	p.R782C	2 M	2	[98]	Carrier: 1 M

Protein domain	Region	Nationality	Mutation Type	Protein Mutation	Sex	Case	Reference	Notes
TKD2	Asia	Japanese	Missense mutation	p.R782C	1 M, 1 NA	2	[37, 39]	
TKD2	Europe	British	Missense mutation	p.R782G	1 M, 2 F	3	[53]	
TKD2	North America	American	Missense mutation	p.R782H	1 M, 7 F	8	[40, 54]	
TKD2	Europe	British	Missense mutation	p.R782H	1 M	1	[25]	
TKD2	Europe	Greek	Missense mutation	p.R782H	1 F	1	[55]	
TKD2	Asia	Chinese	Missense mutation	p.R782H	2 F	2	[56]	Carrier: 1 F
TKD2	Asia	Japanese	Missense mutation	p.R782H	5 M, 2 F, 1 NA	8	[38, 39, 57]	
TKD2	Asia	Korean	Missense mutation	p.R782H	2 M, 2 F	4	[41]	Carrier: 1 M, 1 F
TKD2	Europe	Italian	Missense mutation	p.R782L	1 F	1	[58]	
TKD2	Europe	Croatian	Missense mutation	p.V784M	2 F	2	[59]	
TKD2	Asia	Japanese	Missense mutation	p.A792D	4 M, 1 F, 1 NA	6	[39, 60]	
TKD2	Europe	Polish	Missense mutation	p.A792D	1 M	1	[61]	
TKD2	Asia	Japanese	Missense mutation	p.K793T	2 M, 1 NA	3	[38, 39, 62]	
TKD2	North America	American	Missense mutation	p.I794F	1 M	1	[4]	
TKD2	Europe	Dutchman	Missense mutation	p.I794T	1 M	1	[10]	
TKD2	Asia	Taiwanese	Missense mutation	p.I794T	1 F	1	[63]	
TKD2	North America	American	Missense mutation	p.I794T	3 M, 3 F, 3 NA	9	[4, 15, 16, 64, 65]	
TKD2	Europe	British	Missense mutation	p.I794T	1 M	1	[25]	
TKD2	Asia	Chinese	Missense mutation	p.I794T	6 M, 15 F, 1 NA	22	[1, 26, 34, 66, 67][99]	Carrier: 1 M, 1 F

Protein domain	Region	Nationality	Mutation Type	Protein Mutation	Sex	Case	Reference	Notes
TKD2	Asia	Korean	Missense mutation	p.I794T	1 M, 1 F	2	[68]	
TKD2	Europe	French	Missense mutation	p.I794T	2 M, 1 F	3	[42]	
TKD2	Europe	German	Missense mutation	p.I794T	1 M, 2 F	3	[45, 69]	
TKD2	Asia	Indian	Missense mutation	p.I794T	1 F	1	[70]	
TKD2	Asia	Japanese	Missense mutation	p.I794T	2 M, 5 F, 7 NA	14	[31, 35, 38, 39, 71]	
TKD2	Europe	Irish	Missense mutation	p.I794T	1 M	1	[72]	
TKD2	Asia	Japanese	Missense mutation	p.G798A	1 NA	1	[39]	
TKD2	North America	American	Missense mutation	p.L817P	1 M	1	[10]	
TKD2	North America	American	Missense mutation	p.L817Q	1 M	1	[33]	
TKD2	Asia	Chinese	Missense mutation	p.W821C	1 F	1	[73]	
TKD2	Asia	Chinese	Missense mutation	p.W821R	1 F	1	[1]	
TKD2	Europe	Belgian	Missense mutation	p.M822I	1 F	1	[74]	
TKD2	Asia	Chinese	Missense mutation	p.A823D	2 M	2	[26, 34]	
TKD2	Asia	Japanese	Missense mutation	p.A823V	3 F	3	[21, 75]	
TKD2	Asia	Japanese	Missense mutation	p.P824S	1 F	1	[35]	
TKD2	Europe	Irish	Missense mutation	p.P824R	2 M, 1 F	3	[51]	
TKD2	Europe	British	Missense mutation	p.E825K	1 F	1	[23]	
TKD2	Asia	Japanese	Missense mutation	p.E825K	2 NA	2	[39]	
TKD2	Europe	French	Missense mutation	p.I827T	1 F	1	[10]	
TKD2	North America	American	Missense mutation	p.F828S	2 F	2	[64]	
TKD2	Europe	French	Missense mutation	p.T833K	3 F	3	[42]	

Protein domain	Region	Nationality	Mutation Type	Protein Mutation	Sex	Case	Reference	Notes
TKD2	Asia	Indian	Missense mutation	p.T833M	3 M, 3 F	6	[76]	Carrier: 2 M, 2 F
TKD2	North America	American	Missense mutation	p.D837Y	1 F	1	[15]	
TKD2	Europe	German	Missense mutation	p.V838L	3 M	3	[13, 14, 45]	
TKD2	Europe	French	Missense mutation	p.Y841C	2 F	2	[42]	
TKD2	Asia	Japanese	Missense mutation	p.Y841H	1 NA	1	[39]	
TKD2	Europe	French	Missense mutation	p.G842V	1 M	1	[42]	
TKD2	Europe	German	Del/FS/Dup/Ins	p.I843_L844delinsGI	1 M, 6 F	7	[77]	
TKD2	Europe	Italian	Missense mutation	p.I843F	1 M	1	[30]	
TKD2	Europe	German	Missense mutation	p.I843N	1 F	1	[45]	
TKD2	Asia	Chinese	Missense mutation	p.L845P	1 F	1	[34]	
TKD2	Europe	French	Missense mutation	p.L845P	1 M, 1 F	2	[42]	
TKD2	Europe	British	Missense mutation	p.E847D	1 F	1	[10]	
TKD2	Europe	German	Missense mutation	p.E847D	1 F	1	[78]	
TKD2	Asia	Korean	Missense mutation	p.E847K	1 F	1	[79]	
TKD2	Europe	Italian	Missense mutation	p.E847K	1 M	1	[80]	
TKD2	North America	American	Missense mutation	p.E847V	1 F	1	[81]	
TKD2	North America	American	Del/FS/Dup/Ins	p.F849del	1 F	1	[15]	
TKD2	Asia	Chinese	Del/FS/Dup/Ins	p.F849del	1 F	1	[66]	
TKD2	Europe	British	Missense mutation	p.F849S	1 F	1	[15]	
TKD2	Asia	Taiwanese	Missense mutation	p.F849S	1 F	1	[63]	
TKD2	North America	American	Missense mutation	p.F849S	1 F	1	[15]	

Protein domain	Region	Nationality	Mutation Type	Protein Mutation	Sex	Case	Reference	Notes
TKD2	Asia	Chinese	Missense mutation	p.L851P	2 M, 3 F	5	[26, 82]	Carrier: 2 M, 1 F
TKD2	Europe	Swedish	Missense mutation	p.N854K	9 M, 9 F	18	[83, 84]	Carrier: 2 M, 4 F
TKD2	Asia	Chinese	Missense mutation	p.P855T	5 M, 5 F	10	[66],100]	
TKD2	Europe	French	Missense mutation	p.Y856H	2 M, 4 F	6	[10, 42]	Carrier: 1 M
TKD2	Europe	British	Missense mutation	p.P857L	1 F	1	[25]	
TKD2	North America	American	Missense mutation	p.L868P	1 M, 1 F	2	[15, 17]	
TKD2	Europe	Caucasian	Missense mutation	p.L868R	1 M	1	[9]	
TKD2	North America	Canadian	Missense mutation	p.V869G	1 M	1	[85]	
TKD2	North America	American	Missense mutation	p.M875I	1 M	1	[86]	
TKD2	Asia	Japanese	Missense mutation	p.M875R	1 NA	1	[39]	
TKD2	North America	American	Missense mutation	p.M875T	9 M, 4 F, 2 NA	15	[15, 16]	
TKD2	Europe	German	Nonsense mutation	p.Q877*	2 M	2	[45]	Carrier: 1 M
TKD2	North America	American	Missense mutation	p.P878A	1 M	1	[4]	
TKD2	North America	American	Missense mutation	p.P878S	1 M	1	[4]	
TKD2	North America	American	Missense mutation	p.P878T	1 M, 2 F	3	[15]	
TKD2	Asia	Chinese	Del/FS/Dup/Ins	p.P882Pfs*70	1 M, 1 F	2	[87]	
TKD2	North America	American	Del/FS/Dup/Ins	p.Y886Qfs*55	1 M	1	[4]	
TKD2	Asia	Korean	Del/FS/Dup/Ins	p.C892_A894del	1 M, 1 F	2	[68, 89]	
TKD2	Asia	Chinese	Del/FS/Dup/Ins	p.H899fs	1 M, 2 F	3	[90]	
TKD2	North America	American	Missense mutation	p.R900K	1 NA	1	[91]	
TKD2	Europe	German	Missense mutation	p.R900K	1 M	1	[92]	

Protein domain	Region	Nationality	Mutation Type	Protein Mutation	Sex	Case	Reference	Notes
TKD2	North America	American	Missense mutation	p.P901S	1 NA	1	[10]	
TKD2	Europe	Italian	Missense mutation	p.I906T	1 M	1	[30]	
TKD2	Europe	Caucasian	Missense mutation	p.E916K	1 NA	1	[9]	
TKD2	Europe	Caucasian	Missense mutation	p.E920D	1 NA	1	[9]	
TKD2	Europe	Caucasian	Missense mutation	p.G957R	1 NA	1	[9]	
Other	Asia	Chinese	Del/FS/Dup/Ins	p.F971Sfs*7	2 M	2	[1, 2]	
Other	Asia	Japanese	Del/FS/Dup/Ins	c.889+1G>C	1 M	1	[88]	
Other	North America	American	Del/FS/Dup/Ins	c.1754-1G>C	1 M	1	[27]	Homozygous mutation
Other	Asia	Chinese	Del/FS/Dup/Ins	c.1858+1G>T	2 M, 2 F	4	[93]	
Other	Asia	Chinese	Del/FS/Dup/Ins	c.2319+1C>A	3 M, 1 F	4	[94]	
Other	Asia	Japanese	Del/FS/Dup/Ins	c.2442+1G>A	1 F	1	[101]	
Other	Asia	Japanese	Del/FS/Dup/Ins	c.2442+1G>T	1 M	1	[35]	
Other	Asia	Korean	Del/FS/Dup/Ins	c.2442+1G>T	1 F	1	[95]	
Other	Asia	Japanese	Del/FS/Dup/Ins	c.2442+2T>C	5 M, 1 F	6	[96]	
Other	Asia	Korean	Del/FS/Dup/Ins	c.2442+5G>A	1 F	1	[68]	
Other	Asia	Japanese	Del/FS/Dup/Ins	c.2442+5G>A	1 M, 1 F	2	[19]	
Other	Asia	Chinese	Del/FS/Dup/Ins	c.2442+2_2442+3dupT	1 F	1	[1]	
Other	Asia	Chinese	Del/FS/Dup/Ins	c.2654_2654+3del	2 M, 1 F	3	[67]	
Other	Europe	British	Del/FS/Dup/Ins	c.2655-2A>G	1 F	1	[10]	
Other	Asia	Turkish	Del/FS/Dup/Ins	c.2763+1G>T (chr5:149433884C>A)	1 M, 2 F	3	[97]	Bi-allele mutation
Other	Asia	Chinese	Del/FS/Dup/Ins	delCTC	1 F	1	[66]	

1. Chu M, Wang D-X, Cui Y, Kong Y, Liu L, Xie K-X, et al. Three novel mutations in Chinese patients with CSF1R-related leukoencephalopathy. Annals of Translational Medicine. 2021;9(13):1072-.
2. Wu L, Liu J, Sha L, Wang X, Li J, Dong J, et al. Sporadic Cases with Novel Mutations and Pedigree in Hereditary Leukoencephalopathy with Axonal Spheroids. Journal of Alzheimer's Disease. 2017;56(3):893-8.
3. Giau VV, Senanarong V, Bagyinszky E, An SSA, Kim S. Analysis of 50 Neurodegenerative Genes in Clinically Diagnosed Early-Onset Alzheimer's Disease. Int J Mol Sci. 2019;20(6).
4. Miura T, Mezaki N, Konno T, Iwasaki A, Hara N, Miura M, et al. Identification and functional characterization of novel mutations including frameshift mutation in exon 4 of CSF1R in patients with adult-onset leukoencephalopathy with axonal spheroids and pigmented glia. J Neurol. 2018;265(10):2415-24.
5. Guo L, Bertola DR, Takanohashi A, Saito A, Segawa Y, Yokota T, et al. Bi-allelic CSF1R Mutations Cause Skeletal Dysplasia of Dysosteosclerosis-Pyle Disease Spectrum and Degenerative Encephalopathy with Brain Malformation. Am J Hum Genet. 2019;104(5):925-35.
6. Riku Y, Ando T, Goto Y, Mano K, Iwasaki Y, Sobue G, et al. Early pathologic changes in hereditary diffuse leukoencephalopathy with spheroids. J Neuropathol Exp Neurol. 2014;73(12):1183-90.
7. Monies D, Maddirevula S, Kurdi W, Alanazy MH, Alkhalidi H, Al-Owain M, et al. Autozygosity reveals recessive mutations and novel mechanisms in dominant genes: implications in variant interpretation. Genet Med. 2017;19(10):1144-50.
8. Sassi C, Capozzo R, Hammer M, Zecca C, Federoff M, Blauwendraat C, et al. Exploring dementia and neuronal ceroid lipofuscinosis genes in 100 FTD-like patients from 6 towns and rural villages on the Adriatic Sea cost of Apulia. Sci Rep. 2021;11(1):6353.
9. Sassi C, Nalls MA, Ridge PG, Gibbs JR, Lupton MK, Troakes C, et al. Mendelian adult-onset leukodystrophy genes in Alzheimer's disease: critical influence of CSF1R and NOTCH3. Neurobiol Aging. 2018;66:179 e17- e29.
10. Guerreiro R, Kara E, Le Ber I, Bras J, Rohrer JD, Taipa R, et al. Genetic analysis of inherited leukodystrophies: genotype-phenotype correlations in the CSF1R gene. JAMA Neurol. 2013;70(7):875-82.
11. Chen Z, Tan YJ, Lian MM, Tandiono M, Foo JN, Lim WK, et al. High Diagnostic Utility Incorporating a Targeted Neurodegeneration Gene Panel With MRI Brain Diagnostic Algorithms in Patients With Young-Onset Cognitive Impairment With Leukodystrophy. Front Neurol. 2021;12:631407.
12. Konno T, Miura T, Harriott AM, Mezaki N, Edwards ES, Rademakers R, et al. Partial loss of function of colony-stimulating factor 1 receptor in a patient with white matter abnormalities. Eur J Neurol. 2018;25(6):875-81.
13. Schuberth M, Levin J, Sawalhe D, Schwarzkopf R, von Baumgarten L, Ertl-Wagner B, et al. [Hereditary diffuse leukencephalopathy with spheroids: a microgliopathy due to CSF1 receptor impairment]. Nervenarzt. 2014;85(4):465-70.
14. Hofer TP, Zawada AM, Frankenberger M, Skokann K, Satzl AA, Gesierich W, et al. slan-defined subsets of CD16-positive monocytes: impact of granulomatous inflammation

and M-CSF receptor mutation. Blood. 2015;126(24):2601-10.
15. Rademakers R, Baker M, Nicholson AM, Rutherford NJ, Finch N, Soto-Ortolaza A, et al. Mutations in the colony stimulating factor 1 receptor (CSF1R) gene cause hereditary diffuse leukoencephalopathy with spheroids. Nat Genet. 2011;44(2):200-5.
16. Sundal C, Fujioka S, Van Gerpen JA, Wider C, Nicholson AM, Baker M, et al. Parkinsonian features in hereditary diffuse leukoencephalopathy with spheroids (HDLS) and CSF1R mutations. Parkinsonism Relat Disord. 2013;19(10):869-77.
17. Blauwendraat C, Pletnikova O, Geiger JT, Murphy NA, Abramzon Y, Rudow G, et al. Genetic analysis of neurodegenerative diseases in a pathology cohort. Neurobiol Aging. 2019;76:214 e1- e9.
18. Fujioka S, Broderick DF, Sundal C, Baker MC, Rademakers R, Wszolek ZK. An adult-onset leukoencephalopathy with axonal spheroids and pigmented glia accompanied by brain calcifications: a case report and a literature review of brain calcifications disorders. J Neurol. 2013;260(10):2665-8.
19. Konno T, Broderick DF, Mezaki N, Isami A, Kaneda D, Tashiro Y, et al. Diagnostic Value of Brain Calcifications in Adult-Onset Leukoencephalopathy with Axonal Spheroids and Pigmented Glia. AJNR Am J Neuroradiol. 2017;38(1):77-83.
20. Ho VM, Hovsepian DA, Shieh PB. Myelopathy in a patient with leukodystrophy due to CSF1R mutation. Neurol Genet. 2019;5(6):e376.
21. Abe T, Kawarai T, Fujita K, Sako W, Terasawa Y, Matsuda T, et al. MR Spectroscopy in Patients with Hereditary Diffuse Leukoencephalopathy with Spheroids and Asymptomatic Carriers of Colony-stimulating Factor 1 Receptor Mutation. Magn Reson Med Sci. 2017;16(4):297-303.
22. Daida K, Nishioka K, Li Y, Nakajima S, Tanaka R, Hattori N. CSF1R Mutation p.G589R and the Distribution Pattern of Brain Calcification. Intern Med. 2017;56(18):2507-12.
23. Lynch DS, Jaunmuktane Z, Sheerin UM, Phadke R, Brandner S, Milonas I, et al. Hereditary leukoencephalopathy with axonal spheroids: a spectrum of phenotypes from CNS vasculitis to parkinsonism in an adult onset leukodystrophy series. J Neurol Neurosurg Psychiatry. 2016;87(5):512-9.
24. Bayat M, Shekhrajka N, Bayat A. Hereditary leukodystrophy with axonal spheroids (HDLS) presenting subacutely: a CNS-vasculitis mimic. Acta Neurol Belg. 2019;119(4):633-5.
25. Lynch DS, Rodrigues Brandao de Paiva A, Zhang WJ, Bugiardini E, Freua F, Tavares Lucato L, et al. Clinical and genetic characterization of leukoencephalopathies in adults. Brain. 2017;140(5):1204-11.
26. Tian WT, Zhan FX, Liu Q, Luan XH, Zhang C, Shang L, et al. Clinicopathologic characterization and abnormal autophagy of CSF1R-related leukoencephalopathy. Transl Neurodegener. 2019;8:32.
27. Oosterhof N, Chang IJ, Karimiani EG, Kuil LE, Jensen DM, Daza R, et al. Homozygous Mutations in CSF1R Cause a Pediatric-Onset Leukoencephalopathy and Can Result in Congenital Absence of Microglia. Am J Hum Genet. 2019;104(5):936-47.
28. Zhuang LP, Liu CY, Li YX, Huang HP, Zou ZY. Clinical features and genetic characteristics of hereditary diffuse leukoencephalopathy with spheroids due to CSF1R mutation: a case report and literature review. Ann Transl Med. 2020;8(1):11.
29. Chitu V, Gokhan S, Stanley ER. Modeling CSF-1 receptor deficiency diseases - how close are we? FEBS J. 2021.
30. Battisti C, Di Donato I, Bianchi S, Monti L, Formichi P, Rufa A, et al. Hereditary diffuse leukoencephalopathy with axonal spheroids: three patients with stroke-like presentation carrying new mutations in the CSF1R gene. Journal of Neurology. 2014;261(4):768-72.

31. Mitsui J, Matsukawa T, Ishiura H, Higasa K, Yoshimura J, Saito TL, et al. CSF1R mutations identified in three families with autosomal dominantly inherited leukoencephalopathy. Am J Med Genet B Neuropsychiatr Genet. 2012;159B(8):951-7.
32. Eichler FS, Li J, Guo Y, Caruso PA, Bjonnes AC, Pan J, et al. CSF1R mosaicism in a family with hereditary diffuse leukoencephalopathy with spheroids. Brain. 2016;139(Pt 6):1666-72.
33. Gelfand JM, Greenfield AL, Barkovich M, Mendelsohn BA, Van Haren K, Hess CP, et al. Allogeneic HSCT for adult-onset leukoencephalopathy with spheroids and pigmented glia. Brain. 2020;143(2):503-11.
34. Zhan FX, Zhu ZY, Liu Q, Zhou HY, Luan XH, Huang XJ, et al. Altered structural and functional connectivity in CSF1R-related leukoencephalopathy. Brain Imaging Behav. 2021;15(3):1655-66.
35. Konno T, Tada M, Tada M, Koyama A, Nozaki H, Harigaya Y, et al. Haploinsufficiency of CSF-1R and clinicopathologic characterization in patients with HDLS. Neurology. 2014;82(2):139-48.
36. Du Q, Wang M, Zhou H. A novel mutation in CSF1R associated with hereditary diffuse leukoencephalopathy with spheroids. Neurol Sci. 2021.
37. Konno T, Kasanuki K, Ikeuchi T, Dickson DW, Wszolek ZK. CSF1R-related leukoencephalopathy: A major player in primary microgliopathies. Neurology. 2018;91(24):1092-104.
38. Kinoshita M, Kondo Y, Yoshida K, Fukushima K, Hoshi K, Ishizawa K, et al. Corpus callosum atrophy in patients with hereditary diffuse leukoencephalopathy with neuroaxonal spheroids: an MRI-based study. Intern Med. 2014;53(1):21-7.
39. Kondo Y, Matsushima A, Nagasaki S, Nakamura K, Sekijima Y, Yoshida K. Factors predictive of the presence of a CSF1R mutation in patients with leukoencephalopathy. Eur J Neurol. 2020;27(2):369-75.
40. Nicholson AM, Baker MC, Finch NA, Rutherford NJ, Wider C, Graff-Radford NR, et al. CSF1R mutations link POLD and HDLS as a single disease entity. Neurology. 2013;80(11):1033-40.
41. Kim EJ, Shin JH, Lee JH, Kim JH, Na DL, Suh YL, et al. Adult-onset leukoencephalopathy with axonal spheroids and pigmented glia linked CSF1R mutation: Report of four Korean cases. J Neurol Sci. 2015;349(1-2):232-8.
42. Codjia P, Ayrignac X, Mochel F, Mouzat K, Carra-Dalliere C, Castelnovo G, et al. Adult-Onset Leukoencephalopathy with Axonal Spheroids and Pigmented Glia: An MRI Study of 16 French Cases. American Journal of Neuroradiology. 2018;39(9):1657-61.
43. Makary MS, Awan U, Kisanuki YY, Slone HW. Adult-onset leukoencephalopathy with axonal spheroids and pigmented glia: Clinical and imaging characteristics. Neuroradiol J. 2019;32(2):139-42.
44. Hoffmann S, Murrell J, Harms L, Miller K, Meisel A, Brosch T, et al. Enlarging the nosological spectrum of hereditary diffuse leukoencephalopathy with axonal spheroids (HDLS). Brain Pathol. 2014;24(5):452-8.
45. Karle KN, Biskup S, Schule R, Schweitzer KJ, Kruger R, Bauer P, et al. De novo mutations in hereditary diffuse leukoencephalopathy with axonal spheroids (HDLS). Neurology. 2013;81(23):2039-44.
46. Inui T, Kawarai T, Fujita K, Kawamura K, Mitsui T, Orlacchio A, et al. A new CSF1R mutation presenting with an extensive white matter lesion mimicking primary progressive

multiple sclerosis. J Neurol Sci. 2013;334(1-2):192-5.
47. Yokote A, Ouma S, Takahashi K, Hara F, Yoshida K, Tsuboi Y. [A case of hereditary diffuse leukoencephalopathy with spheroids and pigmented glia presenting with long-term mild psychiatric symptoms]. Rinsho Shinkeigaku. 2020;60(6):420-4.
48. Lubomski M, Buckland ME, Sy J, Wei H, Tan IYL, Kane B, et al. Adult-onset leukoencephalopathy with neuroaxonal spheroids and pigmented glia mimicking systemic lupus erythematosus cerebral vasculitis. J Neurol Sci. 2018;395:25-8.
49. Ahmed R, Guerreiro R, Rohrer JD, Guven G, Rossor MN, Hardy J, et al. A novel A781V mutation in the CSF1R gene causes hereditary diffuse leucoencephalopathy with axonal spheroids. Journal of the Neurological Sciences. 2013;332(1-2):141-4.
50. Prieto-Morin C, Ayrignac X, Ellie E, Tournier-Lasserve E, Labauge P. CSF1R-related leukoencephalopathy mimicking primary progressive multiple sclerosis. J Neurol. 2016;263(9):1864-5.
51. Delaney C, Farrell M, Doherty CP, Brennan K, O'Keeffe E, Greene C, et al. Attenuated CSF-1R signalling drives cerebrovascular pathology. EMBO Mol Med. 2021;13(2):e12889.
52. Saitoh BY, Yamasaki R, Hayashi S, Yoshimura S, Tateishi T, Ohyagi Y, et al. A case of hereditary diffuse leukoencephalopathy with axonal spheroids caused by a de novo mutation in CSF1R masquerading as primary progressive multiple sclerosis. Mult Scler. 2013;19(10):1367-70.
53. Foulds N, Pengelly RJ, Hammans SR, Nicoll JA, Ellison DW, Ditchfield A, et al. Adult-Onset Leukoencephalopathy with Axonal Spheroids and Pigmented Glia Caused by a Novel R782G Mutation in CSF1R. Sci Rep. 2015;5:10042.
54. Robinson JL, Suh E, Wood EM, Lee EB, Coslett HB, Raible K, et al. Common neuropathological features underlie distinct clinical presentations in three siblings with hereditary diffuse leukoencephalopathy with spheroids caused by CSF1R p.Arg782His. Acta Neuropathol Commun. 2015;3:42.
55. Stoiloudis P, Parissis D, Smyrni N, Stardeli T, Afrantou T, Konstantinopoulou E, et al. Hereditary diffuse leukoencephalopathy with spheroids mimicking primary progressive aphasia: report of a Greek case. Neurol Sci. 2021;42(8):3431-3.
56. Shu Y, Long L, Liao S, Yang J, Li J, Qiu W, et al. Involvement of the optic nerve in mutated CSF1R-induced hereditary diffuse leukoencephalopathy with axonal spheroids. BMC Neurol. 2016;16:171.
57. Kinoshita M, Yoshida K, Oyanagi K, Hashimoto T, Ikeda S. Hereditary diffuse leukoencephalopathy with axonal spheroids caused by R782H mutation in CSF1R: case report. J Neurol Sci. 2012;318(1-2):115-8.
58. Bonvegna S, Straccia G, Golfre Andreasi N, Elia AE, Marucci G, Di Bella D, et al. Parkinsonism and Nigrostriatal Damage Secondary to CSF1R-Related Primary Microgliopathy. Mov Disord. 2020;35(12):2360-2.
59. La Piana R, Webber A, Guiot MC, Del Pilar Cortes M, Brais B. A novel mutation in the CSF1R gene causes a variable leukoencephalopathy with spheroids. Neurogenetics. 2014;15(4):289-94.
60. Ueda S, Yamashita H, Hikiami R, Sawamoto N, Yoshida K, Takahashi R. A novel A792D mutation in the CSF1R gene causes hereditary diffuse leukoencephalopathy with axonal spheroids characterized by slow progression. eNeurologicalSci. 2015;1(1):7-9.
61. Konno T, Yoshida K, Mizuno T, Kawarai T, Tada M, Nozaki H, et al. Clinical and genetic characterization of adult-onset leukoencephalopathy with axonal spheroids and pigmented glia associated with CSF1R mutation. Eur J Neurol. 2017;24(1):37-45.

62. Kondo Y, Kinoshita M, Fukushima K, Yoshida K, Ikeda S. Early involvement of the corpus callosum in a patient with hereditary diffuse leukoencephalopathy with spheroids carrying the de novo K793T mutation of CSF1R. Intern Med. 2013;52(4):503-6.
63. Lan MY, Liu JS, Chang CC, Chen YF, Su CS, Peng CH, et al. Clinicopathologic and Genetic Studies of 2 Patients With Hereditary Diffuse Leukoencephalopathy With Axonal Spheroids. Alzheimer Dis Assoc Disord. 2016;30(1):73-6.
64. Kleinfeld K, Mobley B, Hedera P, Wegner A, Sriram S, Pawate S. Adult-onset leukoencephalopathy with neuroaxonal spheroids and pigmented glia: report of five cases and a new mutation. J Neurol. 2013;260(2):558-71.
65. Sharma R, Graff-Radford J, Rademakers R, Boeve BF, Petersen RC, Jones DT. CSF1R mutation presenting as dementia with Lewy bodies. Neurocase. 2019;25(1-2):17-20.
66. Mao C, Zhou L, Zhou L, Yang Y, Niu J, Li J, et al. Biopsy histopathology in the diagnosis of adult-onset leukoencephalopathy with axonal spheroids and pigmented glia (ALSP). Neurol Sci. 2020;41(2):403-9.
67. Xie JJ, Ni W, Wei Q, Ma H, Bai G, Shen Y, et al. New clinical characteristics and novel pathogenic variants of patients with hereditary leukodystrophies. CNS Neurosci Ther. 2020;26(5):567-75.
68. Kim M, Lee H, Cho HJ, Young Chun S, Shin JH, Kim EJ, et al. Pathologic Correlation of Paramagnetic White Matter Lesions in Adult-Onset Leukoencephalopathy With Axonal Spheroids and Pigmented Glia. J Neuropathol Exp Neurol. 2017;76(11):924-8.
69. Meyer-Ohlendorf M, Braczynski A, Al-Qaisi O, Gessler F, Biskup S, Weise L, et al. Comprehensive diagnostics in a case of hereditary diffuse leukodystrophy with spheroids. BMC Neurol. 2015;15:103.
70. Rudrabhatla P, Sabarish S, Ramachandran H, Nair SS. Teaching NeuroImages: Rare Adult-Onset Genetic Leukoencephalopathy. Neurology. 2021;96(20):e2561-e2.
71. Kitani-Morii F, Kasai T, Tomonaga K, Saito K, Mizuta I, Yoshioka A, et al. Hereditary diffuse leukoencephalopathy with spheroids characterized by spastic hemiplegia preceding mental impairment. Intern Med. 2014;53(12):1377-80.
72. Molloy A, Williams L, Farrell M, O'Riordan S. Hereditary Diffuse Leucoencephalopathy and Spheroids Resulting From a Mutation in CSF1R: A Rare Cause of Parkinsonism. Mov Disord Clin Pract. 2014;1(2):132-3.
73. Chen J, Luo S, Li N, Li H, Han J, Ling L. A Novel Missense Mutation of the CSF1R Gene Causes Incurable CSF1R-Related Leukoencephalopathy: Case Report and Review of Literature. Int J Gen Med. 2020;13:1613-20.
74. Coomans C, Sieben A, Lammens M, Ceuterick-de Groote C, Vandenbroecke C, Goethals I, et al. Early-onset dementia, leukoencephalopathy and brain calcifications: a clinical, imaging and pathological comparison of ALSP and PLOSL/Nasu Hakola disease. Acta Neurol Belg. 2018;118(4):607-15.
75. Terasawa Y, Osaki Y, Kawarai T, Sugimoto T, Orlacchio A, Abe T, et al. Increasing and persistent DWI changes in a patient with hereditary diffuse leukoencephalopathy with spheroids. J Neurol Sci. 2013;335(1-2):213-5.
76. Tamhankar PM, Zhu B, Tamhankar VP, Mithbawkar S, Seabra L, Livingston JH, et al. A Novel Hypomorphic CSF1R Gene Mutation in the Biallelic State Leading to Fatal Childhood Neurodegeneration. Neuropediatrics. 2020;51(4):302-6.
77. Kraya T, Quandt D, Pfirrmann T, Kindermann A, Lampe L, Schroeter ML, et al. Functional characterization of a novel CSF1R mutation causing hereditary diffuse leukoencephalopathy with spheroids. Mol Genet Genomic Med. 2019;7(4):e00595.

78. Blume J, Weissert R. Suspected Perinatal Depression Revealed to be Hereditary Diffuse Leukoencephalopathy with Spheroids. J Mov Disord. 2017;10(1):59-61.
79. Kim SI, Jeon B, Bae J, Won JK, Kim HJ, Yim J, et al. An Autopsy Proven Case of CSF1R-mutant Adult-onset Leukoencephalopathy with Axonal Spheroids and Pigmented Glia (ALSP) with Premature Ovarian Failure. Exp Neurobiol. 2019;28(1):119-29.
80. Di Donato I, Stabile C, Bianchi S, Taglia I, Mignarri A, Salvatore S, et al. A Novel CSF1R Mutation in a Patient with Clinical and Neuroradiological Features of Hereditary Diffuse Leukoencephalopathy with Axonal Spheroids. J Alzheimers Dis. 2015;47(2):319-22.
81. Gore E, Manley A, Dees D, Appleby BS, Lerner AJ. A young-onset frontal dementia with dramatic calcifications due to a novel CSF1R mutation. Neurocase. 2016;22(3):257-62.
82. Wang M, Zhang X. A novel CSF-1R mutation in a family with hereditary diffuse leukoencephalopathy with axonal spheroids misdiagnosed as hydrocephalus. Neurogenetics. 2019;20(3):155-60.
83. Granberg T, Hashim F, Andersen O, Sundal C, Karrenbauer VD. Hereditary diffuse leukoencephalopathy with spheroids - a volumetric and radiological comparison with multiple sclerosis patients and healthy controls. Eur J Neurol. 2016;23(4):817-22.
84. Sundal C, Baker M, Karrenbauer V, Gustavsen M, Bedri S, Glaser A, et al. Hereditary diffuse leukoencephalopathy with spheroids with phenotype of primary progressive multiple sclerosis. Eur J Neurol. 2015;22(2):328-33.
85. Lapalme-Remis S, Warman Chardon J, Bourque PR, Oboudiyat C. Diffuse leukoencephalopathy with spheroids presenting as primary progressive aphasia. Neurology. 2016;86(15):1464-5.
86. Tipton PW, Stanley ER, Chitu V, Wszolek ZK. Is Pre-Symptomatic Immunosuppression Protective in CSF1R-Related Leukoencephalopathy? Mov Disord. 2021;36(4):852-6.
87. Du Q, Chen H, Shi Z, Zhang Y, Wang J, Zhou H. A novel mutation in the CSF1R gene causes hereditary diffuse leukoencephalopathy with axonal spheroids. Neurol Sci. 2019;40(6):1287-90.
88. Kunii M, Doi H, Ishii Y, Ohba C, Tanaka K, Tada M, et al. Genetic analysis of adult leukoencephalopathy patients using a custom-designed gene panel. Clin Genet. 2018;94(2):232-8.
89. Kim EJ, Kim YE, Jang JH, Cho EH, Na DL, Seo SW, et al. Analysis of frontotemporal dementia, amyotrophic lateral sclerosis, and other dementia-related genes in 107 Korean patients with frontotemporal dementia. Neurobiol Aging. 2018;72:186 e1- e7.
90. Shi T, Li J, Tan C, Chen J. Diagnosis of hereditary diffuse leukoencephalopathy with neuroaxonal spheroids based on next-generation sequencing in a family: Case report and literature review. Medicine (Baltimore). 2019;98(22):e15802.
91. Cochran JN, McKinley EC, Cochran M, Amaral MD, Moyers BA, Lasseigne BN, et al. Genome sequencing for early-onset or atypical dementia: high diagnostic yield and frequent observation of multiple contributory alleles. Cold Spring Harb Mol Case Stud. 2019;5(6).
92. Kortvelyessy P, Krageloh-Mann I, Mawrin C, Heinze HJ, Bittner D, Wieland I, et al. Hereditary diffuse leukoencephalopathy with spheroids (HDLS) with a novel CSF1R mutation and spinal cord involvement. J Neurol Sci. 2015;358(1-2):515-7.
93. Yang X, Huang P, Tan Y, Xiao Q. A Novel Splicing Mutation in the CSF1R Gene in a Family With Hereditary Diffuse Leukoencephalopathy With Axonal Spheroids. Front Genet. 2019;10:491.

94. Leng C, Lu L, Wang G, Zhang Y, Xu Y, Lin X, et al. A novel dominant-negative mutation of the CSF1R gene causes adult-onset leukoencephalopathy with axonal spheroids and pigmented glia. Am J Transl Res. 2019;11(9):6093-101.
95. Lee D, Yun JY, Jeong JH, Yoshida K, Nagasaki S, Ahn TB. Clinical evolution, neuroimaging, and volumetric analysis of a patient with a CSF1R mutation who presented with progressive nonfluent aphasia. Parkinsonism Relat Disord. 2015;21(7):817-20.
96. Kawakami I, Iseki E, Kasanuki K, Minegishi M, Sato K, Hino H, et al. A family with hereditary diffuse leukoencephalopathy with spheroids caused by a novel c.2442+2T>C mutation in the CSF1R gene. J Neurol Sci. 2016;367:349-55.
97. Kindis E, Simsek-Kiper PO, Kosukcu C, Taskiran EZ, Gocmen R, Utine E, et al. Further expanding the mutational spectrum of brain abnormalities, neurodegeneration, and dysosteosclerosis: A rare disorder with neurologic regression and skeletal features. Am J Med Genet A. 2021;185(6):1888-96.
99. Bai Y, Lu L, Cui Y, et al. Analysis of clinical and neuroimaging features in a Chinese family with hereditary diffuse leukoencephalopathy with neuroaxonal spheroids. Chin J Neurol 2018; 51:877-81.
100. Cheng X, Shen W, Zou H, Shen L, Gu X, Huang D, Sun Y, Wang B, Tian Q & Xu J. Analysis of CSF1R gene mutation in a Chinese family with hereditary diffuse leukoencephalopathy with neuroaxonal spheroids. Zhonghua Yi Xue Yi Chuan Xue Za Zhi 2015; 32, 208–212.
101. Saitoh B-Y, Yoshida K, Hayashi S, Yamasaki R, Sato S, Kamada T, Suzuki SO, Murai H, Iwaki T, Ikeda SI et al. Sporadic hereditary diffuse leukoencephalopathy with axonal spheroids showing numerous lesions with restricted diffusivity caused by a novel splice site mutation in the CSF1R gene. Clin Exp Neuroimmunol 2013; 4, 7 6–81.
