# Supplementary material for: Insights Into the Role of CSF1R in the Central Nervous System and Neurological Disorders
Source: Front Aging Neurosci. 2021 Nov 15;13:789834. doi: 10.3389/fnagi.2021.789834 (PMC8634759; doi:10.3389/fnagi.2021.789834)
Supplement: Supplementary file 2 [file Table_2.pdf]

Supplementary Table 2. Nationality distribution of *CSF1R* mutations in ALSP.

Region	Nationality	Number of Case (s)	Percentage
America (110)	American	107	23.3%
	Brazilian	2	
	Canadian	1	
Asia (232)	Arab	8	49%
	Chinese	90	
	Japanese	94	
	Turkish	3	
	Indian	7	
	Korean	20	
	Thailander	2	
	Taiwanese	2	
	Caucasian	1	
	Chaldean	5	
Europe (131)	Swedish	18	27.7%
	Belgian	1	
	Polish	1	
	Italian	7	
	German	31	
	British	19	
	Dutchman	2	

	Greek	3	
	Danish	1	
	French	26	
	Irish	8	
	Norwegian	4	
	Caucasian	8	
	Croatian	2	
Total		473	100%
