# Supplementary material for: Insights Into the Role of CSF1R in the Central Nervous System and Neurological Disorders
Source: Front Aging Neurosci. 2021 Nov 15;13:789834. doi: 10.3389/fnagi.2021.789834 (PMC8634759; doi:10.3389/fnagi.2021.789834)
Supplement: Supplementary file 3 [file Table_3.pdf]

Supplementary Table 3. Location of *CSF1R* variants in CSF1R protein domain.

Protein domain	Number of Mutation Type	Percentage	Number of Case (s)	Percentage
Signal peptide	1	0.7%	2	0.4%
EC	6	4.3%	9	1.9%
TM	1	0.7%	1	0.2%
JMD	7	5.0%	9	1.9%
TKD1	22	15.7%	76	16.1%
KID	6	4.3%	6	1.3%
TKD2	83	59.3%	337	71.2%
Other	14	10.0%	33	7.0%
Total	140	100%	473	100%

TKD：75.0%（Mutation of type）；87.3% (Number of cases)
