# Supplementary material for: Insights Into the Role of CSF1R in the Central Nervous System and Neurological Disorders
Source: Front Aging Neurosci. 2021 Nov 15;13:789834. doi: 10.3389/fnagi.2021.789834 (PMC8634759; doi:10.3389/fnagi.2021.789834)
Supplement: Supplementary file 4 [file Table_4.pdf]

Supplementary Table 4. The effectiveness of the CSF1R inhibitors on microglia depletion, amyloid/tau pathology and synaptic function in the text.

CSF1R inhibitor	Percentage of Microglial depletion	Amyloid /tau pathology	Synaptic function	Mouse model	References
PLX5622	Extensive microglial depletion	Reduced plaques in the visual cortex	Prevent perineuronal nets (PNNs) loss	5×FAD	Crapser et al., 2020
	Low dose: 30% microglia	Prevent microglial association with plaques	NA	3×Tg-AD	Dagher et al., 2015
	90%	Impair parenchymal plaque development	NA	5×FAD	Spangenberg et al., 2019
	Almost completely (1200 mg/kg) ablated	Not alter plaque load	Less effective in altering synaptic function	App^{NL-F} and App^{NL-G-F}	Benitez et al., 2021
	over 50%	Reduce plaque burden	Enhance neuritic dystrophy	5×FAD	Casali et al., 2020
	Almost completely absent	Increase plaque deposition; reduce propagation of p-tau	NA	App^{NL-G-F}	Clayton et al., 2021
PLX3397	～80%	Not mediate or protect from amyloid pathology	Prevent neuronal loss	5×FAD	Spangenberg et al., 2016
	～81% reduction of microglial area in the cortex	Attenuation of amyloid-facilitated tau pathology	NA	Heterozygous mice of 5×FAD and P301S	Lodder et al., 2021
	30%	Not affect tau pathology	No change in neuron loss or count	Tg4510	Bennett et al., 2018
	70–80%	Reduce plaque deposition	NA	5×FAD	Sosna et al., 2018
JNJ-527	40%	Attenuation of tau pathology	Reduction of neuronal cell death	P301S	Mancuso et al., 2019

**GW2580**	85%	Reduce the Aβ plaques	Reduction in the axonal pathology	APP/PS1	Hu et al., 2021
	Significant reduction	Improve memory performance but not change amyloid-β plaques	Prevent synaptic degeneration	APP/PS1	Olmos-Alonso et al., 2016

Notes: NA, not available. All references are listed in the main text.
